# B cell lymphoma 6 promotes hepatocellular carcinoma progression by inhibiting tumor infiltrating CD4^+^T cell cytotoxicity through ESM1

**DOI:** 10.1038/s41698-024-00625-7

**Published:** 2024-07-01

**Authors:** Jiatao Li, Juan Feng, Ziyong Li, Yuanli Ni, Limei Liu, Xia Lei, Zixuan Chai, Na Zhuang, Jiake Xu, Yongpeng He, Juanjuan Shan, Cheng Qian

**Affiliations:** 1 Chongqing Key Laboratory of Translational Research for Cancer Metastasis and Individualized Treatment, Chongqing University Cancer Hospital, Chongqing, 400030 China; 2 School of Medicine Chongqing University, Chongqing, 400030 China

**Keywords:** Immunosurveillance, Oncogenes

## Abstract

Immunotherapy exhibited potential effects for advanced hepatocellular carcinoma, unfortunately, the clinical benefits are often countered by cancer adaptive immune suppressive response. Uncovering the mechanism how cancer cells evade immune surveillance would help to develop new immunotherapy approaches and combination therapy. In this article, by analyzing the transcriptional factors which modulate the differentially expressed genes between T cell infiltration high group and low group, we identified oncoprotein B cell lymphoma 6 (BCL6) suppresses the infiltration and activation of tumor infiltrating T lymphocytes, thus correlated with poorer clinical outcome. By using antibody deletion experiment, we further demonstrated that CD4^+^T cells but not CD8^+^T cells are the main lymphocyte population suppressed by Bcl6 to promote HCC development. Mechanistically, BCL6 decreases cancer cell expression of pro-inflammatory cytokines and T lymphocyte chemokines such as IL6, IL1F6, and CCL5. Moreover, BCL6 upregulates Endothelial cell-specific molecule 1 (ESM1) to inhibit T lymphocyte recruitment and activation possibly through ICAM-1/LFA-1 signaling pathway. Our findings uncovered an unappreciated paracrine mechanism how cancer cell-derived BCL6 assists cancer cell immune evasion, and highlighted the role of CD4^+^T cells in HCC immune surveillance.

## Introduction

Hepatocellular carcinoma (HCC) accounts for 75–85% of primary liver cancer cases and is the fourth leading cause of cancer-related deaths worldwide^1,2^. Since HCC is difficult to diagnosis until late in the disease, HCC patients often progress to late stage where local treatments or resection cannot be performed^3^. Cancer neo-antigen makes it possible for immunotherapy to enhance immune system defenses against cancer cells. Indeed, advances in immunotherapy-based treatment strategies such as targeting programmed cell death protein 1(PD-1) and cytotoxic T-lymphocyte-associated antigen 4 has brought new hope for the treatment of advanced HCC^4^. However, in clinic only few patients respond to immune checkpoint inhibitor (ICI) therapy while other patients do not respond or develop resistance. The low response rate and drug resistance limited the efficacy of immunotherapy^5^. The mechanisms of cancer cell immune evasion include extrinsic mechanisms like immunosuppressive cells in tumor microenvironment as well as intrinsic signaling pathways such as decreased antigen presentation ability, loss of PD-L1 expression^6^. Uncovering new mechanism how cancer cell modulate the immune microenvironment would provide new strategy to re-boost the cytotoxicity of lymphocyte against cancer cells.

Most immune therapeutic modalities focused on improving the quantity and quality of antitumor CD8^+^ cytotoxic T lymphocyte response, however, the role of CD4^+^T cells in antitumor response remain largely elusive. Studies have reported that CD4^+^T cells inhibit tumor progression in three ways, including secreting inflammatory factors to promote the cytotoxicity of NK cells, myeloid cells and CD8^+^T cells; affecting the function of B cells to assist the production of specific antibodies; and directly recognizing and killing tumor cells through MHCII^7^. The fundamental role of CD4^+^T cells in anti-tumor process was further enhanced by the finding that immunogenic tumor mutations in the “mutanomes” of three separate preclinical mouse tumor models induced a CD4^+^T cell response but not a CD8^+^T cells response as had been expected^8^. Furthermore, Bastian Kruse et al. uncovered that CD4^+^T cells and microenvironment myeloid cells orchestrate the induction of remote inflammatory cell death that indirectly eradicates interferon-unresponsive and MHC-deficient tumors^9^. This observation extends to CAR T cell adoptive immunotherapy, which showed that CD4^+^CAR T cells have anti-tumor effector activity independent of CD8^+^CAR T cells^10^. Nevertheless, whether CD4^+^T cells also play a role in HCC immune surveillance and how cancer cells suppress CD4^+^T cell function was not well studied.

B-cell lymphoma 6 (BCL6) is a transcriptional repressor that belongs to the BTB/POZ family of transcription factors. It was widely studied as a master regulator of humoral immunity, where BCL6 plays critical role in the initiation and maintenance of the germinal center^11^. In addition, BCL6 has also been reported as a proto-oncogene in lymphoma, acute lymphoblastic leukemia, acute myeloid leukemia, and solid cancers including breast cancer^12^, glioma^13^ and non-small cell lung cancer^14^ by suppressing genes involved in high-fidelity DNA repair, cell differentiation and promotes cell proliferation, anti-apoptosis, cell-cycle arrest, which are the hallmarks of cancer^15,16^. The function of BCL6 in modulating immune cell function such as follicular T cell and B cell function is largely attributed to its direct role in modulating T or B cells. Whether cancer cell derived BCL6 participates in cancer immune evasion and whether BCL6 plays a role in HCC progression has not been studied.

Herein, by analyzing the transcriptional factors which modulate differentially expressed genes between T cell infiltration high patient group and low group from TCGA data base, we found that BCL6 is a potential immune suppressor for hepatocellular carcinoma. With further in vivo and in vitro experiments, we demonstrated that BCL6 promoted HCC evasion from CD4^+^T cells mediated cytotoxicity. Mechanistically, BCL6 suppress pro-inflammatory cytokines expression by HCC cancer cells and promoted the expression of immune suppressive protein ESM1, which was reported to be antagonist of ICAM-1 in binding to T cell surface protein LFA-1. Our research expanded the mechanism of BCL6 in cancer progression, and highlighted the role of CD4^+^T cells in HCC immune surveillance.

## Results

### Transcription factor analysis suggests that BCL6 is a potential immunosuppressive transcriptional factor in HCC

**Fig. 1:**
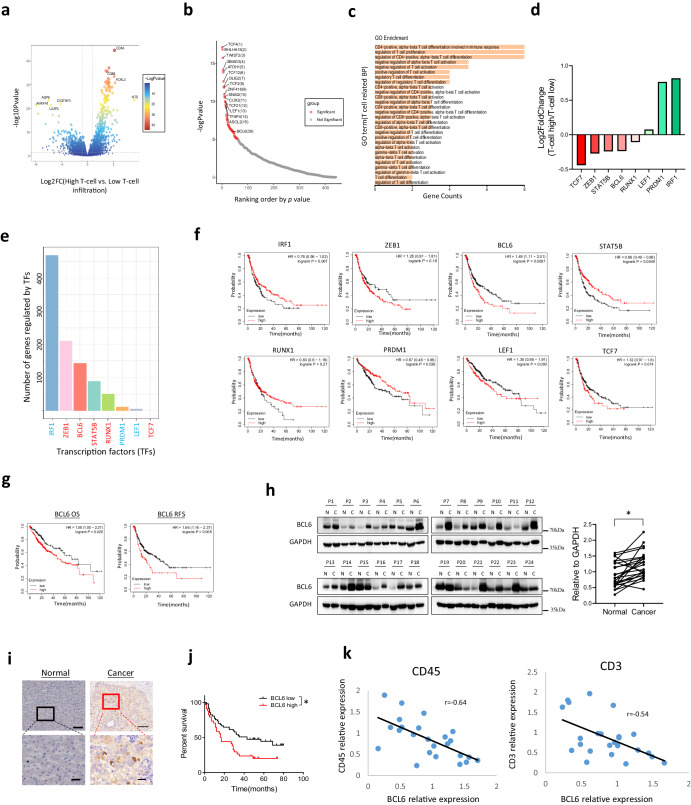
BCL6 expression is upregulated in HCC cancer tissue and is correlated with poor clinical outcome. **a** Volcano plot for differentially expressed genes between T-cell high infiltration group and T-cell low infiltration group. **b** Transcription factors ranked by *p* value. **c** GO_BP pathway analysis for the TFs. **d** Fold change of the eight candidate TFs between T-high and T-low groups involved in T cell function as shown in (**c**). **e** Number of genes regulated by the 8 candidate TFs within the differentially expressed genes between T-high and T-low groups. **f** Progression-free survival data for the 8 TFs. **g** Overall survival and Recurrence-free survival for BCL6. **h** Western blot and quantification for BCL6 in HCC cancer tissue and the adjacent normal tissue, N: adjacent normal tissue, C: HCC cancer tissue, P: patient. **i** Immunohistochemistry for BCL6 in HCC cancer and adjacent normal tissue. **j** Survival curve based on the HCC samples from immunohistochemistry. **k** Correlation of CD45, CD3 expression with BCL6 expression based on western blotting for HCC patient samples. * *p* < 0.05; scale for the upper panel of immunohistochemistry is 100 µm, lower panel is 20 µm.

T cells are the main population of immune cells account for cancer immune rejection, thus immune check point blockade therapy which aims to reboost the function of T cells achieved fundamental progress for cancer treatment. To find the key modulator for T cell infiltration and activation, we utilized LM22 leukocyte gene signature matrix, which contains 547 genes that distinguish 22 human hematopoietic cell phenotypes including T cells, neutrophils, B cells, macrophages et al, to analyze immune cell infiltraiton in 374 HCC samples from The Cancer Genome Atlas (TCGA). We grouped them according to the quartile of T cell infiltration score. Top 25% T cell infiltration samples were defined as T-cell high group, while lower 25% T cell infiltration samples were defined as T-cell low group. Then the differential expressed were compared between T-cell high and T-cell low groups using DESeq2 method. As shown in Fig. 1a, about 1977 differentially expressed genes with |log2FC| > 0.6 and *p* value < 0.05 were found. To find which transcriptional factors (TFs) modulate the expression of these genes, we next predict the potential transcription factors by performing a motif analysis using HOMER. Promoter regions of the differential genes were enriched for 61 TFs (Fig. 1b).

GO_BP pathway analysis further indicated that 8 genes including TCF7, ZEB1, STAT5B, BCL6, RUNX1, LEF1, PRDM1, and IRF1 are associated with T cell infiltration, activation and differentiation (Fig. 1c). TCF7, ZEB1, STAT5B, BCL6 and RUNX1 are downregulated in T-cell high group, while LEF1, PRDM1 and IRF1are upregulated in T-cell high group, indicating that TCF7, ZEB1, STAT5B, BCL6, RUNX1 potentially suppress T cell function, while LEF1, PRDM1, IRF1 promote T cell activation (Fig. 1d). Figure 1e demonstrated the number of differential genes (T-cell high compared to T-cell low) regulated by each TFs indicated by hTFtarget database. By analyzing these TFs association with HCC patient clinical outcome including overall survival (OS), Progression-free survival (PFS) and Recurrence free survival using KM-plot^17^, we found BCL6 was associated with worse clinical outcome compared to other 7 TFs (Fig. 1f, g and Fig. S1a, S1b).

As a highly conserved transcript suppressor, BCL6 was reported to act as proto-oncogene by affecting cancer hallmark pathways including proliferation, DNA repair, anti-apoptosis, and enable cancer cells to adapt to stress in both lymphoma and solid tumor. However, its role in HCC progression and the underlying mechanism is largely unknown. To analysis the expression of BCL6 in HCC tissue, we firstly applied data from The Cancer Genome Atlas (TCGA) to determine the pan-cancer expression of BCL6 in different cancer types. We found that BCL6 is elevated in many cancer types including GBM, KIRC, SARC and LIHC (*P* = 0.0786) (Fig. S1c). To confirm the expression in protein level, we used western blot to analyze BCL6 expression in HCC patient samples and the related adjacent normal tissue. In accordance with TCGA data, western blotting revealed that for most HCC samples, BCL6 was elevated in the cancer tissue compared to the normal adjacent tissue (Fig. 1h). We also applied immunohistochemistry to visualize the location of BCL6 in HCC and found that high expression of BCL6 was located in cancer cells but not in the stromal cells (Fig. 1i). Based on the BCL6 expression from immunohistochemistry, we divided HCC patients into BCL6 high and BCL6 low groups. By analyzing the overall survival time, we found that in accordance with KM-plot data, high expression of BCL6 was correlated with poor patient clinical outcome (Fig. 1j). Since in bioinformatics analysis we showed that BCL6 is negatively correlated with T cell infiltration, to confirm this conclusion, we used western blot to analyze BCL6 expression and immune cell infiltration (CD45) as well as T cell infiltration (CD3). From the Pearson correlation analysis based on western blotting expression data, we observed that BCL6 is indeed negatively correlated with CD45expression (*r* = −0.64) and CD3 expression (*r* = −0.54) (Fig. 1k).

Conclusively, these data suggested that liver cancer cells express higher level of BCL6 compared with normal hepatocytes, and the expression of BCL6 in HCC tissue is correlated with poor clinical outcome.

### Bcl6 promotes HCC progression

**Fig. 2:**
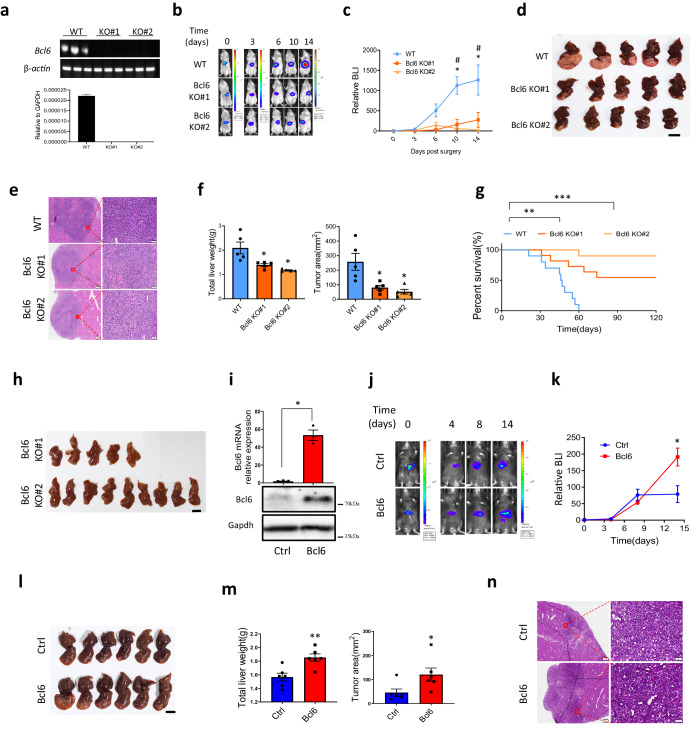
BCL6 promotes HCC progression. **a** Analyze the knockout efficiency of H22 Bcl6 knockout lines #1 and #2 using qRT-PCR. **b** In vivo imaging for H22 tumor growth after orthotopic injection in liver capsule. **c** HCC tumor growth curve quantified from in vivo imaging, *n* = 5 per group. **d** Liver image for H22 derived liver tumor, scale for liver images: 10 mm. H&E staining, left panel scale: 500μm, right panel scale: 50 μm (**e**) and quantification of the liver weight as well as tumor area (**f**) for H22 derived liver tumor, *n* = 5 per group. **g** Mice survival curve after injection of H22 WT or Bcl6 KO, *n* = 10 per group. **h** Liver image for the survived mice at 120 days post H22 injection from the growth curve shown in (**g**). **i** Confirmation of Bcl6 overexpression efficiency in Hepa1-6 with qPCR and western blot, *n* = 3 for the qPCR data. **j** In vivo imaging for Hepa1-6 ctrl and Bcl6 overexpression. **k** Tumor growth curve quantified from in vivo imaging data derived from (**j**), *n* = 6 per group. **l** Liver image for the mice after injection of Hepa1-6 at day 14 post surgery and quantification of total liver weight as well as tumor area (**m**), *n* = 6 per group. **n** H&E confirmation of the cancer tissue derived from (**l**), left panel scale: 500 μm, right panel scale: 50 μm. * *p* < 0.05, ** *p* < 0.01, ***p < 0.001.

Role of BCL6 in other cancer types such as breast cancer were mostly studied in immune deficient mice, however, this mouse model could not well simulate the function of BCL6 in human cancer progression, where immune cells play fundamental role in modulating tumor growth. Thus, to study the role of BCL6 in liver cancer progression, we used mouse cell lines H22 and Hepa1-6 which were derived from BALB/c and C57 respectively. To demonstrate the necessity of Bcl6 in liver cancer progression, we constructed two BCL6 knockout cell lines using CRISPR/Cas9 technology in H22, and the knockout efficiency of BCL6 was confirmed using quantitative PCR (qPCR) as shown in Fig. 2a as well as genomic PCR results (Fig. S2). In vitro proliferation assay using both CCK8 and growth curve data both suggested that knockout of Bcl6 slightly decreased H22 proliferation (Fig. S3a, S3b).

Next, to study Bcl6 function in vivo, H22 cells line was labeled with luciferase using protocol as shown previously^18^, thus we could use in vivo imaging system (IVIS) to monitor the tumor growth in a real-time manner. We injected H22 cells orthotopically in liver capsule and applied IVIS to monitor tumor growth from day0 to day14 post-surgery as shown in Fig. 2b. By normalizing the bioluminescence imaging (BLI) signal at each time point to day0 initial signal, we found that Bcl6 knockout significantly decreased HCC progression (Fig. 2c). Since H22 is very malignant and the tumor grows very fast, we sacrificed the mice on day14 post-surgery. From the liver image in Fig. 2d we can see that in accordance with BLI signal, knocking out of Bcl6 decreased tumor growth, which as further confirmed by H&E staining (Fig. 2e) as well as quantification of total liver weight and tumor area (Fig. 2f). Furthermore, we used survival curve to analyze the effect of Bcl6 on tumor progression in longer time period and found that knocking out of Bcl6 significantly extended mice survival time (Fig. 2g). Notably, when we sacrificed the survived mice on day 120 post-surgery for BCL6 KO#1 and KO#2 groups, we found that all the remaining mice were tumor free (Fig. 2h).

To confirm the function of BCL6 in liver cancer progression, we overexpressed Bcl6 in Hepa1-6 (Fig. 2i), which is a more mild HCC cell line compared to H22. As in knockout experiment, we labeled Hepa1-6 with luciferase so that we could check the growth using in vivo imaging. Bcl6 overexpression efficiency was analyzed using qPCR and western blot (Fig. 2i). We also checked the effect of Bcl6 overexpression on Hep1-6 growth in vitro and found that in accordance with knockout data, Bcl6 overexpression mildly increased the growth of Hep1-6 (Fig. S3c). In vivo experiments showed that overexpression of BCL6 did not alter HCC progression before day 7. However, on day 14 post surgery, control Hepa1-6 group signal did not alter significantly compared to day 7, which may possibly be due to immune surveillance, while overexpression group progress much faster compared to control (Fig. 2j, k). We sacrificed the mice on day 14, and the liver image, liver weight, tumor area as well as the H&E staining all supported that overexpression of Bcl6 promoted liver cancer progression (Fig. 2l–n). To ask whether the properties of tumor in transplant experiment is the same as the original cell line, we used qPCR to analyse the differentiation-related genes including Afp, Albumin and K19, as well as liver cancer stem cell-related genes such as Prom1, Thy-1, Nanog, and Anpep. From Fig. S4 we found that the tumor expresses higher levels of Afp, Albumin as well as Prom1, Thy-1 and Anpep for both wild-type H22 or Bcl6 knockout cell lines derived tumors than the corresponding cell lines (Fig. S4).

Collectively, in this part we used in vivo function experiment to show that Bcl6 promotes HCC progression.

### Single cell RNA sequencing revealed BCL6 knockout turned “cold” tumor into “hot” tumor

**Fig. 3:**
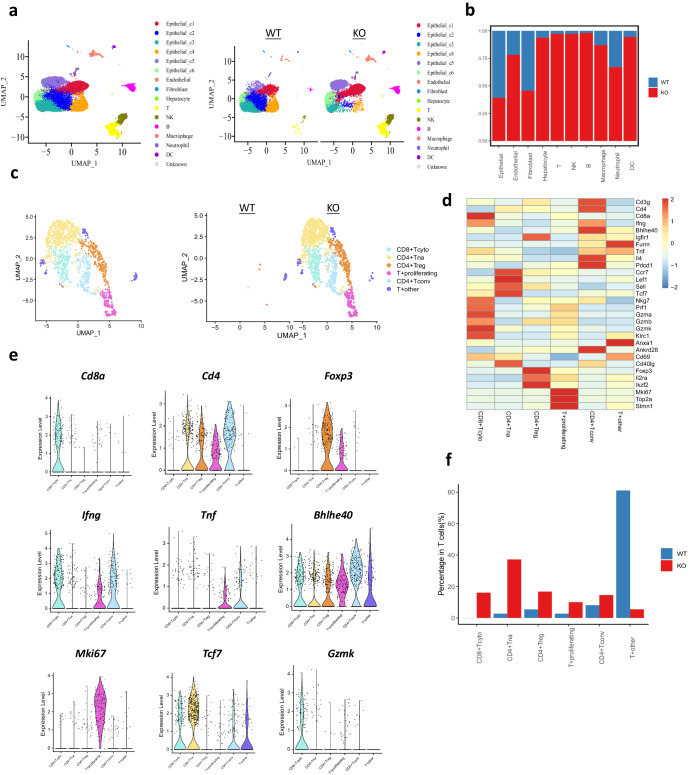
Single-cell RNA sequencing reveals that BCL6 turned “cold” tumor into “hot” tumor. **a** UMAP clusters for wild type and Bcl6 knockout H22 derived tumor. **b** Percentage of each cell population derived from H22 WT and Bcl6 KO tumors. **c** UMAP for T cell re-clustering, T+proliferating: proliferating T cells, T+other: undefined other T cells. **d** Heatmap for marker gene expression for T cell clusters. **e** Violin plot of representative marker gene expression for each T cell cluster. **f** T cell subpopulation distribution for WT and KO groups.

To uncover the mechanism how Bcl6 affects liver cancer progression, we applied single-cell RNA to analyze the expression of tumor cells and infiltrating immune cells. On day 14 post seeding of the cancer cells, we harvested the tumor and digested into single cells, and then sort the alive cells for single cell RNA sequencing using 10×genomics platform. After quality control and dimension reduction using UMAP method, same number of cells from each group were combined together and the total cells were separated into 16 groups based on the marker gene reported (Fig. S5a) including cancer cell cluster 1-6, endothelial, fibroblast, hepatocyte, CD4^+^T, NK cell, B cell, macrophage, neutrophil, DC cells as shown in Fig. 3a. The violin plot for the marker gene expression for both immune and non-immune cells were shown in Fig. S5b, S5c. We can conclude from Fig. 3a that cancer cells from control group formed different clusters compared with KO group, indicating the difference in gene expression after Bcl6 knockout. In addition, there were more immune cells infiltrated in Bcl6 KO group such as innate NK cells, macrophages as well as lymphocytes including T cells and B cells (Fig. 3a). We quantified the percentage of cells from each group for every cell type, in accordance with the functional phenotype as indicated in previous part, we indeed found more cancer cells in the wild type (WT) group (Fig. 3b). In addition, more than 90% of T cells, NK cells and B cells were from Bcl6 KO group, and there are also more infiltration of other immune cells in KO group (Fig. 3b).

Besides the increase of immune cells after Bcl6 knockout, we asked whether the state of the immune cells was also affected. Given the important role of T cells in cancer surveillance, we focused our further analysis on T cells. We sorted T cells out, then used resolution 0.5 for T cells dimension reduction. We obtained five T cell populations, including CD8^+^cytotoxic T cells, CD4^+^T naïve, CD4^+^Treg, proliferating T cell, CD4^+^T convention helper cells, and other T cells (Fig. 3c). The marker genes used for T cell cluster were shown in Fig. 3d. Violin plot showed that Ifnγ, Tnfα, Gzmk, which are needed for T cell cytotoxicity were enriched in cytotoxic CD8^+^T cells (CD8^+^T cyt) and CD4^+^ conventional helper T cells (CD4^+^T conv) population. Proliferating T cells also express high level of Ifnγ and Tnfα, indicating they may also possess tumor cytotoxicity (Fig. 3e). High expression of proliferation marker mKi67 was enriched in proliferating T cells population and also expressed in some T cells from other T populations (Fig. 3e). By quantify the T cell subpopulation distribution for WT and Bcl6 KO groups, we found that about 80% WT group derived T cells located in T other population, which express much lower level of tumor cytotoxic genes. While there was higher percentage of cytotoxic CD8^+^T cells and T convention helper cells for Bcl6 knockout group (Fig. 3f). Collectively, from these data, we can conclude that Bcl6 knockout turned the immune cells deficient “cold” tumor into cytotoxic immune cells rich “hot” tumor.

### Bcl6 suppresses T-cell infiltration and activation

**Fig. 4:**
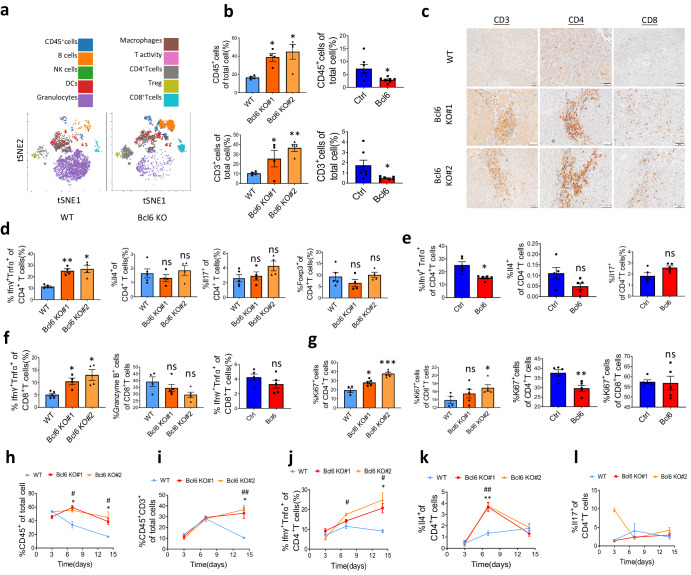
BCL6 inhibits T-cell infiltration and activation. **a** tSNE distribution of immune cells after dimensional reduction for CyTOF of H22 WT and Bcl6 KO tumors. **b** CD45^+^immune cells and CD45^+^CD3^+^T cells quantification using FACS for H22 tumors and Hepa1-6 tumors, *n* = 4–6 per group. **c** Immunohistochemistry showed more infiltration of T cells in H22 Bcl6 KO-derived tumor compared to WT, scale: 50 μm. **d** FACS quantification of Th1, Th2, Th17 and Treg for H22 WT and Bcl6 KO tumors, *n* = 4 per group. **e** FACS quantification of Th1, Th2, Th17 and Treg for Hepa1-6 vector (Ctrl) and Bcl6 overexpression (Bcl6) tumors, *n* = 4 per group. **f** FACS quantification of IfnR^+^Tnfa^+^CD8^+^T cells and Granzyme B^+^CD8^+^T cells infiltration in H22 derived liver tumors and Hepa1-6 derived tumors, *n* = 4 per group. **g** Flow cytometry quantification of CD4^+^and CD8^+^T cells proliferation, *n* = 4 per group. FACS quantification shown the CD45^+^immune cells (**h**), CD45^+^CD3^+^T cells (**i**), Th1 (**j**), Th2 (**k**) and Th17 (**l**) infiltration kinetics in H22 WT and Bcl6 KO liver tumors, *n* = 3–4 per group each time point, *: Bcl6 KO#1 compared to WT, #: Bcl6 KO#2 compared to WT. *p < 0.05, **p < 0.01, ***p < 0.001, #*p* < 0.05, ##*p* < 0.01, ns: not significant.

To confirm single RNA sequencing data which indicated more infiltration of immune cells infiltration after Bcl6 knockout, we applied mass cytometry (CyTOF) to analyzed immune cells in the liver tumor. As shown in Fig. 4a, we can see that within CD45^+^ immune cells, lymphocytes including B cells, CD4^+^T cells and CD8^+^T cells were elevated compared to control group, while innate percentage in Bcl6 knockout group decreased. Then, we used Fluorescence-activated Cell Sorting (FACS) to quantify the immune cell infiltration. In accordance with the single cell RNA sequencing data, total CD45^+^ percentage significantly increased after Bcl6 knockout, while overexpression of Bcl6 decreased immune cell infiltration (Fig. 4b). Total CD3^+^T cells infiltration also increased after knocking out of Bcl6 and decreased after Bcl6 overexpression (Fig. 4b). Immunohistochemistry against CD3, CD4 and CD8 further confirmed more CD4^+^T and CD8^+^T cells infiltration in Bcl6 knockout liver tumors (Fig. 4c).

Above data only showed quantity of T cells infiltration increased in Bcl6 knockout tumor, but whether the infiltrated T cells are activated as indicated in single-cell RNA sequencing data was still not unclear. So we analyzed the state of the T cells by using flow cytometry for T cell differentiation as well as T cell exhaustion markers (Fig. S6). Pro-inflammatory Th1 which was marked by the expression of Ifnγ and Tnfα increased significantly in Bcl6 knockout group while Th2 (Il4^+^), Th17 (Il17^+^) or Treg (Foxp3^+^) percentage did not alter compared to WT control (Fig. 4d). Accordingly, Th1 decreased after Bcl6 overexpression and Th2 and Th17 did not change (Fig. 4e). The Ifnγ^+^Tnfα^+^CD8^+^T cells percentage of total CD3^+^T cells also increased in Bcl6 knockout group, but the Granzyme B^+^CD8^+^T cells are comparable (Fig. 4f). However, after Bcl6 overexpression, the Ifnγ^+^ Tnfα^+^CD8^+^T cells percentage did not change (Fig. 4f). T cell proliferation, another indicative for T cell activation, also increased after Bcl6 knockout (Fig. 4g). Notably, CD8^+^T cell proliferation did not decrease significantly after Bcl6 overexpression, suggesting that the effect of Bcl6 on CD8^+^T cell activation is much less prominent compared to CD4^+^T cells (Fig. 4g). Interestingly, T cell exhaustion, marked by the expression of PD1 and Tim3, was not affected by Bcl6 (Fig. S7a, S7b), which indicates that Bcl6 does not affect T cell exhaustion in HCC tumor.

To ask at which stage the tumor infiltrating T cell infiltration and activation has been affected by Bcl6, we used flow cytometry to analyze CD45^+^ immune cells and T cells infiltrating kinetics on day 3, day 7 and day 14 post-surgery. At early time point day 3, no significantly more immune cells were observed after Bcl6 knockout. However, CD45^+^immune cells increased significantly on day7 for Bcl6 KO group (Fig. 4h). Total T cell infiltration did not alter within the first week, while increased significantly in Bcl6 KO group compared to WT control (Fig. 4i). The elevation of Ifnγ^+^ Tnfα^+^CD4^+^T cells occurred as early as day 7 but become more prominent on day14 (Fig. 4j). Th2 cells were significantly increased on day7 but dropped to equal level on day14 (Fig. 4k). Th17 has no difference between the groups at any time point (Fig. 4l). In addition to T cells, we also analyzed innate immune cells and found that NK cells and Ly6C^+^CD11b^+^MDSC significantly increased after Bcl6 knockout (Fig. S7c, S7d), while macrophages and Ly6G^+^CD11b^+^MDSC were not affected (Fig. S7e, S7f).

### CD4^+^ but not CD8^+^T cells are the main lymphocyte population responsible for Bcl6 knockout induced tumor regression

**Fig. 5:**
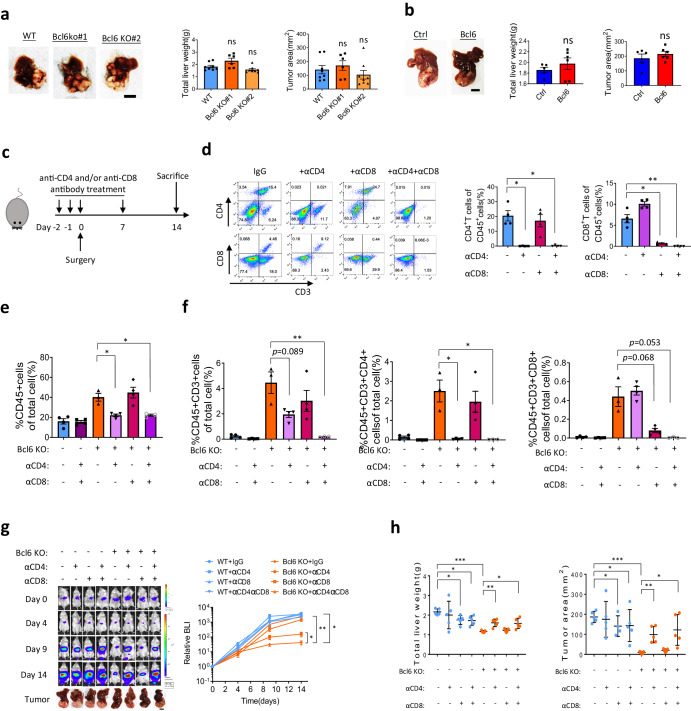
BCL6 suppresses CD4^+^ T cells mediated tumor immune surveillance. **a** Liver tumor image, liver weight and tumor volume after transplantation of H22 in nude mice for 2 weeks, *n* = 7–8. **b** Liver tumor image, quantification of liver weight and tumor volume after transplantation of Hepa1-6 in nude mice for 1 month, *n* = 5–6. **c** Schematic of experiment design. **d** Flow cytometry for blood T cell depletion efficiency and specificity at 2 weeks after treatment with anti-CD4 (aCD4) and/or anti-CD8 (aCD8) antibodies, *n* = 4 per group. Flow cytometry quantification of H22-derived tumor immune cell infiltration (**e**) and T cell infiltration (**f**) after antibody treatment, *n* = 3–4 per group. **g** In vivo imaging as well as liver cancer images for H22 derived tumor growth after antibodies treatment, *n* = 5 per group. **h** Quantification of total liver weight and tumor area after treatment of depletion antibodies at 2 weeks post-surgery, *n* = 5 per group. *p < 0.05, **p < 0.01, ***p < 0.001, ns: not significant, tumor image scale: 10 mm.

We have demonstrated that Bcl6 knockout tumor has more T cell infiltration and the infiltrating T cell activation was also upregulated after Bcl6 knockout. However, whether T cell immune surveillance suppressed tumor growth or the tumor volume is accounted for T cell infiltrating difference is not answered. In addition, as previously described, NK cell infiltration, which is reported to suppress cancer progression, is also suppressed by Bcl6. Whether T cells is the immune population regulated by Bcl6 to promote tumor progression and which subpopulation of T cells is accounted for HCC regression is not answered. So in this part, we firstly repeated Bcl6 function using in vivo experiment in immune deficient nude mice, which do not have functional T cells but have other immune cells including B cells, NK cells, and macrophages. Interestingly, unlike the phenotype observed in immune competent mice, tumor growth was no longer affected by Bcl6 knockout or overexpression in nude mice (Fig. 5a, b). Therefore, we can conclude that Bcl6 inhibited T cells mediated tumor rejection.

To further ask which subpopulation of T cells were inhibited by Bcl6 to promote tumor immune evasion, we used CD4^+^T and CD8^+^T cell-specific depletion antibody to study the function of Bcl6 as shown in Fig. 5c. The specificity and depletion efficiency of these antibodies on CD4^+^T cells and CD8^+^T cells was confirmed with FACS in mouse peripheral blood. Anti-CD4 and anti-CD8 antibody depletion efficiency reached more than 90%, and they would not affect the percentage of the CD8^+^T cells and CD4^+^T cells, respectively (Fig. 5d). On day14 post-surgery, we sacrifice the mice and analyzed the infiltration of immune cells in liver tumors after depletion of CD4^+^T cells and/or CD8^+^T cells. Tumor infiltrating CD45^+^immune cells was decreased after CD4^+^T cells depletion but not after CD8^+^T cells depletion (Fig. 5e). Interestingly, immune cells percentage in single treatment of anti-CD4 antibody group dropped to anti-CD4 and anti-CD8 group, indicating that CD4^+^T cells induced tumor inflammation. Depletion efficiency and specificity of anti-CD4 antibody and anti-CD8 antibody on tumor infiltrating T cells were confirmed in Fig. 5f.

After the confirmation of T cell depletion specificity and efficiency, we used in vivo imaging to monitor the tumor growth in these groups. From Fig. 5g we can see that, depletion of CD4^+^ and/or CD8^+^ T cells would not affect the growth of wild-type H22 liver cancer, which may be due to the severely immune suppressive tumor microenvironment. However, after Bcl6 knockout, depletion of CD4^+^T cells could rescue the tumor growth to the level which is comparable to combined depletion of CD4^+^T cells and CD8^+^T cells group, while depletion of CD8^+^T cells could only slightly increase tumor growth (Fig. 5g). This conclusion was also confirmed by the total liver weight and tumor area on day 14 (Fig. 5h).

### Bcl6 inhibits cancer cell pro-inflammatory pathways and upregulates the expression of T cell co-stimulation suppressor Esm1

**Fig. 6:**
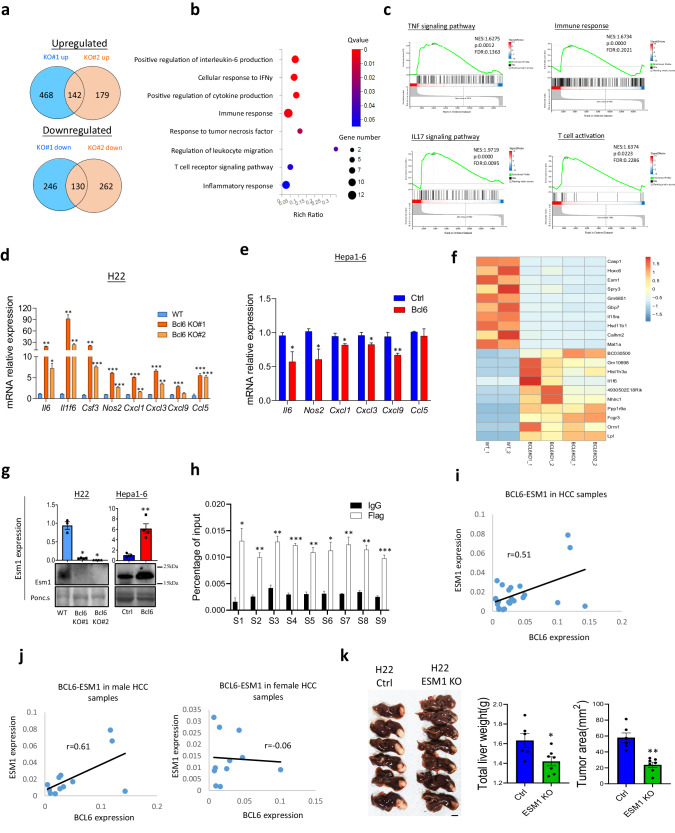
BCL6 inhibits cancer cell inflammatory cytokines secretion and immune suppressive gene expression. **a** Venn diagram for Bcl6 KO#1 and #2 mutually up- or down-regulated genes. **b** GO_P enriched pathways after depletion of Bcl6. **c** GSEA enriched immune related pathways in Bcl6 knockout group. **d** qPCR for immune cytokines in H22 WT vs Bcl6 KO, and Hepa1-6 ctrl vs Bcl6 overexpression (**e**), *n* = 3 per group. **f** Heatmap for top 10 genes been upregulated and downregulated after Bcl6 knockout. **g** qPCR and western blot showing that Bcl6 promoted Esm1 expression, *n* = 3. **h** Chromatin Immunoprecipitation (ChIP) indicated that Bcl6 binds directly to *Esm1* gene promoter. **i** Pearson correlation data for ESM1 expression and BCL6 expression in HCC total patient samples based on qPCR data. **j** Pearson correlation data for ESM1 expression and BCL6 expression in HCC male and female patient samples based on qPCR data. **k** Liver cancer images and quantification of tumor area and liver weight in H22 WT and H22 ESM1 KO groups, *n* = 6–7 per group (scale: 10 mm). *p < 0.05, **p < 0.01.

To find out the downstream potential target proteins which account for Bcl6-mediated tumor immune evasion, we applied bulk RNA sequencing to elucidate the gene transcription after Bcl6 knockout. Firstly, we used Venn diagram to find the mutually upregulated and downregulated genes in Bcl6 KO#1 and KO#2 groups. As shown in Fig. 6a, by using logFC>1 and *q* value < 0.05 as cut off, we found 142 upregulated genes and 130 downregulated genes after Bcl6 knockout. Then, based on the differential expressing genes, GO_P analysis revealed pathways related to immune response. IL-6 signaling pathway and IFNγ response were most significantly enriched in Bcl6 knockout cancer cells (Fig. 6b). GSEA enrichment data further demonstrated that Bcl6 knockout increased the activation of immune cell-related pathways including TNF signaling pathway, IL17 signaling pathway, immune response and T cell activation related pathway (Fig. 6c). We used qPCR to confirm the expression of the immune-related genes involved in immune activation as revealed in RNA sequencing data. From Fig. 6d, pro-inflammatory cytokines including Il6, Csf3, Il1f6 et al., which were reported to be pro-inflammatory proteins, and the immune cell chemokines including Cxcl1, Cxcl3, Cxcl9, and Ccl5 were indeed upregulated after Bcl6 knockout (Fig. 6d). In addition, overexpression of Bcl6 also suppressed these pro-inflammatory genes expression unless some genes not been detected in Hepa1-6 cells (Fig. 6e).

In addition to be a gene suppressor, Bcl6 was also reported to promote genes expression in a direct or indirect way^13^. From the RNA sequencing data, we observed that Bcl6 knockout indeed decreased genes expression (Fig. 6a). We have demonstrated the top10 up or down-regulated genes in Fig. 6f, and found that besides suppressing pro-inflammatory cytokines or chemokines as shown in Fig. 6d, f, Bcl6 knockout also decreased the expression of inflammatory suppressive genes endothelial-cell specific molecule-1 (Esm1), which ranked as top 3 among all downregulated genes.

ESM1 is a 50 kDa cysteine-rich secreted proteoglycan and reported to suppress T cell adhesion, migration and activation through suppression of co-stimulation pathway^19^. In normal tissues, ESM1 is secreted by endothelial cells, lung submucosal glands et al. However, its expression was elevated in many cancer types including lung cancer^20^, renal cancer^21^, liver cancer^22^ and breast cancer^23^. In addition, previous reports using Meta-analysis indicated that ESM1 is associated with poorer overall survival of HCC patients and could be a useful biomarker for patients with hepatocellular carcinoma^24^. Indeed, from TCGA data we found that ESM1 was upregulated in many cancer types compared to normal tissue including hepatocellular carcinoma (Fig. S8a), we also confirmed the increased ESM1 expression in HCC sample with our HCC samples (Fig. S8b). High ESM1 expression was associate with worse clinical outcome for the patients with HCC (Fig. S8c), indicating its crucial function in HCC progression. Further analysis revealed that the expression of Esm1 in both mRNA and protein levels were decreased following Bcl6 knockout, while its expression was increased after Bcl6 overexpression (Fig. 6g).

Bcl6 was reported to upregulate the expression of oncogene through direct binding to target gene promoter^13^, thus we used chromatin immunoprecipitation, coupled with quantitative real-time PCR (qPCR) to prove whether Bcl6 could directly promote Esm1 expression. From Fig. 6h, Bcl6 was indeed recruited in the promoter of Esm1 (Fig. 6h). To ask whether BCL6 could also regulate ESM1 in human, we used qPCR to analyze the expression correlation of BCL6 and ESM1. As demonstrated in Fig. 6i, BCL6-ESM1 Pearson correlation value is 0.51. Dana Lau-Corona et al. reported that gene expression is sexually biased in liver cancer, to ask whether BCL6 regulation of ESM1 is sex-biased, we analyzed the correlation of BCL6 and ESM1 in male and female HCC samples. Interestingly, BCL6-ESM1 correlation is significant in male (*r* = 0.61) but not in female patients (*r* = −0.06) (Fig. 6j). To show the function of ESM1 in liver cancer progression, we knocked out ESM1 in H22 and analyzed tumor progression (Fig. S9). From Fig. 6k we found that knockout of ESM1 decreased H22 progression, partially recapitulate the function of Bcl6.

Collectively, in this part we have shown that knockout of Bcl6 increased HCC cancer cell expression of pro-inflammatory genes while decreasing the expression of anti-inflammatory gene expression including ESM1, and ESM1 promotes liver cancer progression.

### Esm1 suppresses T cell function and combined targeting of BCL6 and PD1 improved immunotherapy efficiency

**Fig. 7:**
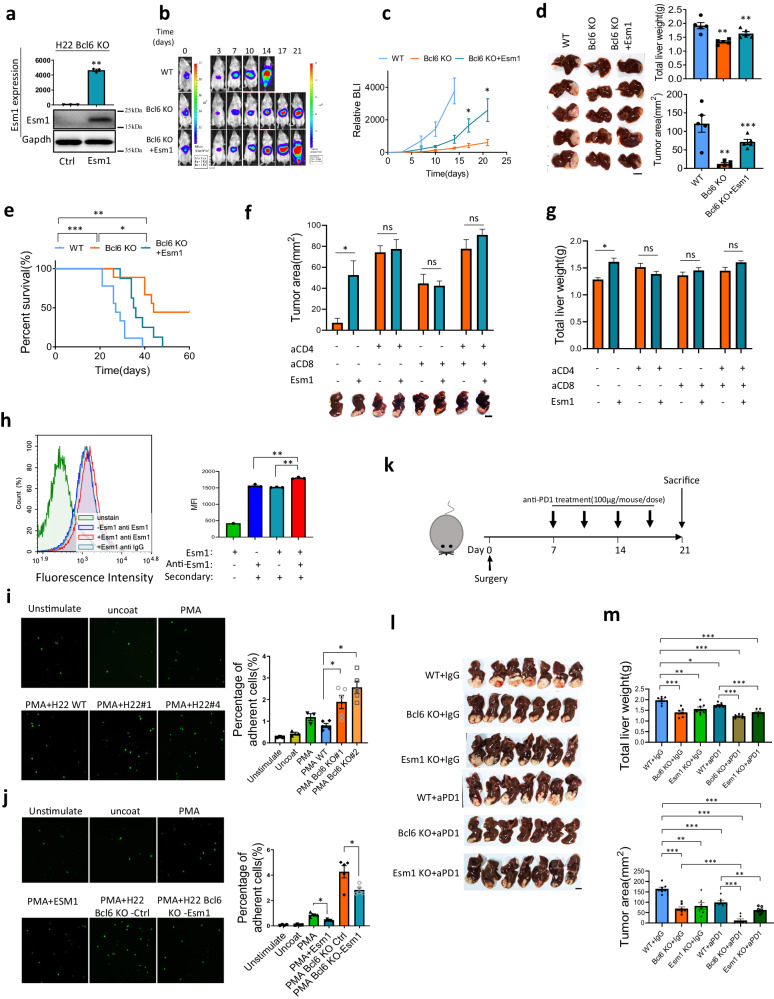
ESM1 suppresses T cell activation possibly through inhibiting T cell ICAM-1/LFA-1 co-stimulation pathway. **a** qPCR and western blot for Esm1 overexpression efficiency, *n* = 3 for qPCR quantification. **b** In vivo imaging for H22 WT, Bcl6 KO and Bcl6 KO+Esm1 overexpression. **c** H22 derived liver cancer growth curve based on in vivo imaging data, *n* = 5 per group. **d** H22 derived liver tumor image from IVS experiment and quantification of liver weight as well as tumor area, *n* = 5 per group. **e** Survival curve for H22 WT, Bcl6 KO and Bcl6 KO+Esm1 overexpression, *n* = 10 per group. Tumor images and tumor area (**f**) as well as total liver weight (**g**) quantification for H22 Bcl6 KO with or without overexpression of Esm1 at 3 weeks post-surgery (aCD4:anti-CD4, aCD8:anti-CD8). **h** Flow cytometry indicated that Esm1 binds directly to CD4^+^T cell surface, *n* = 3 per group for MFI quantification. **i** ICAM-1 adhesion experiments indicated that condition medium from H22 Bcl6 KO enhanced CD4^+^T cell adhesion to ICAM-1, unstimulated: CD4^+^T cells without activation with PMA, PMA WT: PMA activated T cells treated with H22 wild type condition medium, PMA Bcl6 KO#1: PMA activated CD4^+^T cells treated with H22 Bcl6 KO#1 condition medium, PMA Bcl6 KO#2: PMA activated CD4^+^T cells treated with H22 Bcl6 KO#2 condition medium, *n* = 3 per group. **j** Esm1 abolished Bcl6 KO enhanced CD4^+^T cells adhesion to ICAM-1, PMA+Esm1: PMA activated CD4^+^T cells were treated with Esm1 recombinant protein, *n* = 3 per group. **k** Schematic graph for the in vivo experiment. Liver images (**l**) and quantification of tumor area as well as liver weight (**m**) for the anti-PD-1 or IgG treated mice (aPD1: anti-PD1), *n* = 7 per group. *p < 0.05, **p < 0.01, ***p < 0.001, ns: not significant, tumor image scale: 10 mm.

In order to ask whether Esm1 mediates the function of Bcl6, we overexpressed Esm1 in Bcl6 knockout cells. qPCR and western blot confirmed the overexpressing efficiency of Esm1 as shown in Fig. 7a. Then, we used in vivo imaging to monitor tumor growth, and found that Esm1 could partially rescue the phenotype of Bcl6 knockout (Fig. 7b, c). Tumor image, tumor weight and tumor area further supported the conclusion (Fig. 7d). Survival curve also showed that Bcl6 knockout prolonged the survival compared to wild type control, while overexpression of Esm1 decreased the survival compared to Bcl6 knockout group, even though not to be comparable to WT control group (Fig. 7e). Notably, we also used in vitro growth curve to show that overexpression of Esm-1 did not affect cancer cell proliferation (Fig. S10a).

Bcl6 knockout increased total immune cell infiltration, T cell infiltration, and T cell activation as shown previously; we thus used flow cytometry to analyze the tumor immune cells infiltration. We found that Esm1 overexpression decreased CD45^+^ immune cell and CD3^+^ T cell infiltration compared to Bcl6 KO group, but was still higher than WT control group (Fig. S10b), while the Ifnγ^+^Tnfα^+^ CD4^+^T cells and CD8^+^T infiltration in Esm1 overexpression group dropped to be comparable of the wild type control group (Fig. S10c). These data demonstrated that overexpression of Esm1 suppressed immune cell infiltration and T-cell activation. Next, we used CD4 and/or CD8-specific depletion experiment to ask whether Esm1 promotes HCC progression through CD4^+^T cells or CD8^+^T cells. We treated the mice with the depleting antibodies described previously, then analyzed tumor growth at 3 weeks post-surgery. From both tumor area and liver weight we found that Esm1 overexpression rescued tumor growth. However, depletion of either CD4^+^ or CD8^+^T cells abolished the function of Esm1 on tumor growth (Fig. 7f, g). Therefore, both CD4^+^ and CD8^+^T cells are required for Esm1 to promote HCC progression. We also tested the function of BCL6 overexpression on inflammatory gene expression in mouse liver cancer cell line Hep53.4 and human liver cancer cell line Hep3B with qPCR, which both supported that BCL6 overexpression suppressed inflammatory genes expression which promote ESM1 expression (Fig. S11a, S11b). We further tested the correlation of BCL6 expression with inflammatory genes found that BCL6 is negatively correlated with IL1F6, CSF3, but positively correlated with CXCL1, CCL5, NOS2 as well as ESM1. In addition, we analyzed the correlation in male and female groups separately and found that BCL6-ESM1 correlation is most significant in male (*r* = 0.61) but not in female (*r* = −0.06) (Fig. S11c, S11d).

Human ESM1 binds directly to LFA-1 onto the cell surface of human blood lymphocytes, monocytes, and Jurkat cells, thus inhibited the specific binding of ICAM-1 to Jurkat cells^19^. Therefore, ESM1 could be implicated in the regulation of the LFA-1/ICAM-1 pathway and affect lymphocytes recruitment, adhesion and activation. Since we demonstrated in vivo that mouse Esm1 inhibited HCC T cell infiltration and activation, we asked whether Esm1 mediated T cell activation through ICAM-1/LFA-1 pathway. Firstly, we used flow cytometry to demonstrate that Esm1 could directly bind to T cell surface (Fig. 7h). Then by using ICAM-1 adhesion assay, we found that cancer cell-derived conditioned medium from Bcl6 knockout group increased T cell adhesion to ICAM-1 coated plate compared to wild type cancer medium (Fig. 7i). Esm1 recombinant protein decreased T cell adhesion to ICAM-1, and cancer cell medium from Esm1 overexpression group inhibited T cell adhesion compared to Bcl6 knockout control group (Fig. 7j). These data suggested that Esm1 mediate T cell function through mediating T cell ICAM-1/LFA-1 signaling pathway. Collectively, we found Bcl6 upregulate Esm1 to suppress T cell migration and activation, which could be through affecting ICAM-1/LFA-1 signaling pathway.

Whether the immune suppressive effect of BCL6 as well as its downstream target ESM1 could enhance immunotherapy efficiency? To address this question, we used anti-PD1 immune checkpoint inhibitor to test BCL6 and ESM1 function with the in vivo protocol as shown in Fig. 7k. At 3 weeks post-surgery, we sacrificed the mice and found that BCL6 knockout significantly enhanced anti-PD1 efficiency (Fig. 7l, m). Importantly, there were four out of seven mice (about 57%) been tumor free for the BCL6 knockout in combination with anti-PD1 therapy. However, ESM1 knockout has minimal effect on anti-PD1 treatment (not significant) (Fig. 7l, m).

## Discussion

BCL6 was reported to act as proto-oncogene by affecting cancer hallmark pathways including proliferation, DNA repair, anti-apoptosis, and enable cancer cells to adapt to stress^25^. As a highly conserved protein, BCL6 play nonredundant role in immune modulation. In GC formation, B cells derived BCL6 inhibited T cell immune recognition of highly mutated B cells, while BCL6 is also a marker gene for T follicular helper CD4 cells (Tfh), which are key for the induction and maintenance of humoral immune response^26^; in cancer immunology, cancer infiltrating Treg require BCL6 to exert its immune suppressive function^25^. In hepatocyte-specific Bcl6 knockout mouse model, Chikada et al. showed that Bcl6 knockout attenuated choline-deficient, L-amino-acid-defined, high-fat diet (CDAHFD) induced NASH and suppressed NASH-derived tumor progression^27^, implying a role of Bcl6 in liver lipid metabolism and lipoprotein transport. However, the exact mechanism how Bcl6 affects hepatocellular carcinoma progression was not well elucidated. Here in HCC, high expression Bcl6 was observed in HCC cancer cells but not in the microenvironment cells and correlated with poorer clinical outcome (Fig. 1). Further, we used xenograft mouse model to show that Bcl6 in cancer cell has mild effect on tumor growth in immune-deficient mice, but could prominently increase tumor progression in immune-competent mice, suggesting a role of Bcl6 in cancer immune evasion (Figs. 2, 4). Indeed, from single cell RNA sequencing and flow cytometry data, we demonstrated that cancer cells with high expression of Bcl6 inhibit tumor immune cell infiltration, and suppress tumor-infiltrating CD4^+^T cells and CD8^+^T cells activity (Fig. 3). Interestingly, using antibody depletion experiments, we found that only CD4^+^T cell deletion but not CD8^+^T cells deletion aborted T lymphocyte cytotoxicity against HCC after Bcl6 knockout in cancer cells (Fig. 5). Therefore, we conclude that CD4^+^T cells play an important role in HCC immune surveillance, and cancer cell-derived Bcl6 inhibited CD4^+^T cell function to assist HCC immune evasion. Mechanistically, by using bulk RNA sequencing to study the cancer cell gene expression, we demonstrated that Bcl6 downregulated HCC inflammatory-related genes expression including Il1f6, Il6, Csf3, Ccl5 et al.; as well as upregulated immune suppressive protein including Esm1 (Fig. 6).

Previous studies of ESM-1 in inflammatory showed contradictory results. Bechard et al. used in vitro experiment to demonstrate that ESM1 directly bind to integrin CD11a/CD18 (LFA-1), which is expressed on mononuclear cells and important for leukocyte adhesion and activation. The binding of ESM-1 to LFA1 inhibited the specific binding of ICAM-1 to Jurkat cells, implying that ESM1 could regulate LFA-1/ICAM-1 pathway and thus suppress lymphocyte recruitment to inflammatory sites and activation^19^. However, in vivo experiments and clinical data both supported that ESM1 promotes inflammatory and account for inflammatory related disease including ventilator associated pneumonia^28^, cardiovascular disease^29^, and sepsis^30^. By using Esm1 knockout mice Rocha et al. showed that Esm1 does not affect leukocyte rolling and adhesion in vivo, but positively regulates leukocyte extravasation at the endothelial transmigration level^31^. These results implied ESM1 plays opposite role in inflammatory response. Later, using mass spectrometry, Nathalie De Freitas Caires et al. found that neutrophil derived Cathepsin G cleaves Esm1 into a 14 kDa fragment (p14)^32^. p14 could bind to LFA-1 thus inhibit the interaction between LFA-1 and Esm1, and because p14 does not have an inhibition effect on ICAM-1/LFA-1 binding, the competitive binding of p14 and Esm1 with ICAM-1 in turn leads to the restoration of the ICAM-1/LFA-1 interaction^33^. Therefore, Esm1 fragment p14 acts a pro-inflammatory role by acting as antagonistic of Esm1. With these evidence, we hypothesis that whether Esm1 plays a pro-inflammatory or anti-inflammatory role is tissue and microenvironment dependent. Here in hepatocellular carcinoma, we used in vivo experiments to demonstrate that cancer cells express Esm1 to inhibit CD4^+^T cells mediated cancer cytotoxicity, which supports the anti-inflammatory role of ESM1 in HCC development. These conclusion suggested the potential benefit of using ESM1 neutralizing antibodies to inhibit tumor immune evasion.

In this study, we focused on the mechanism that BCL6 inhibits the tumor infiltrating CD4^+^T cells through ESM1, however, there are other mechanisms through which BCL6 promotes HCC progression. First, the effect of BCL6 on HCC cancer cell proliferation also contribute to HCC development. In vitro cell CCK8 experiment and growth curve both revealed that overexpression of Bcl6 significantly improved the proliferation rate of HCC cells, while knockdown of Bcl6 reduced HCC proliferation (Fig. S3). The tumor image, tumor area and total liver weight in nude mice also implied a trend that BCL6 knockout slightly decreased HCC progression, while overexpression of Bcl6 upregulated HCC growth in nude mice (Fig. 5a, b). However, by comparing the effect of Bcl6 on HCC progression in immunocompetent mice (Fig. 2) and immune-deficient mice (Fig. 5a, b), it is reasonable to conclude that the effect of BCL6 on HCC progression through immune microenvironment is much more prominent than its effect on cancer cell growth. Second, from the single-cell RNA sequencing data (Fig. 3b), CyTOF data as well as the flow cytometry results (Fig. 4), we can tell that in addition to T cells, other innate immune cells percentage was also upregulated upon Bcl6 knocking out including NK cells, which was reported to eliminate cancer cells in HCC^34^. Therefore, the function of BCL6 on the innate immune cells may also play a role in HCC progression. In addition, since ICAM-1/LFA-1 signaling pathway is also important for innate immune cell function including macrophages^35^, the Bcl6-Esm1 axis may also mediate innate immunity in HCC microenvironment. Third, from the bulk RNA sequencing data, pro-inflammatory including Il6, Il1f6, and Ccl5 et al. are upregulated after Bcl6 knockout, as their effect on immune cell activation were well studied in other articles, we did not discuss their function in Bcl6 mediated CD4^+^T cell function in this study. Notably, the upregulated inflammatory genes after BCL6 knockout may help to explain why overexpression of Esm1 only partially rescued Bcl6 knockout effect on HCC progression and immune cell infiltration. Lastly, from Fig. 4f, g we demonstrated that Bcl6 also inhibited CD8^+^T cell infiltration and activation. For function, we also found that even though CD4^+^T cells have higher cytotoxicity than CD8^+^T cells towards HCC, blockade of CD8 also improved cancer cell progression in Bcl6 KO group (Fig. 5g). Therefore, even though the effect of Bcl6 on HCC progression was largely attributed to inhibition the function of CD4^+^T cells, the suppressive role of Bcl6 on CD8^+^T cell also partially contributed to HCC progression.

Collectively, we uncovered an unappreciated paracrine mechanism of how Bcl6 promotes cancer progression and highlighted the role of CD4^+^T cells in HCC immune surveillance. We also proved that Esm1 is downstream of Bcl6, and could mediate T lymphocyte immune suppression, thus provide new target and potential diagnostic marker for HCC diagnosis and treatment.

## Methods

### Tissue samples

For immunohistochemistry staining of BCL6 in human samples and the patient survival correlation with BCL6 expression, a tissue microarray of Chinese HCC was purchased from Shanghai Outdo Biotech Co., Ltd (Shanghai, China) (The sample information was shown in Supplementary Table 1). For the western blot of BCL6 expression in HCC, fresh HCC specimens were obtained with written informed consent from patients according to protocols approved by Chongqing University Cancer Hospital. The research proposal was approved by the Ethics Committee of Chongqing University Cancer Hospital (No. CZLS2022022-A) (The sample information was shown in Supplementary Table 2). Human sample collection procedures have complied with all relevant ethical regulations including the Declaration of Helsinki.

### Bioinformatics analysis for TCGA samples

374 HCC samples from The Cancer Genome Atlas (TCGA) were downloaded and analyzed with CIBERSORT^36–39^ to compare the gene expression feature sets of 22 immune cell subtypes based on leukocyte signature matrix (LM22). According to the quartile score of T cells for each sample, top 25% T cell infiltration samples were defined as T-cell high group, lower 25% T cell infiltration samples were defined as T-cell low group. Differentially expressed genes (DEGs) between T-cell high and T-cell low groups were evaluated using DESeq2. False discovery rate (FDR) was used to identify the threshold of the p-value in multiple tests in order to compute their significant differences. Only genes with |log2 (FoldChange)| ≥ 0.6 and an FDR significance score (*p*_adj_) < 0.05 were included in the subsequent analysis^40^.

Motif enrichment analysis for each DEGs was handled by HOMER, a software used for motif discovery and next-gen sequencing analysis. Analysis was performed with the program findMotifs.pl on promoter regions defined between 2000 bp upstream and 2000 bp downstream of each gene’s major isoform TSS. *Q* values were adjusted using Benjamini-Hochberg method, and predicted TFs with *q* value > 0.05 were filtered. TFs were sorted and plotted by the value of “−log *P* value”^41^.

For GO enrichment analysis, we used org.Hs.eg.db (version 3.18.0) in R software for gene GO annotation as the background. The differential genes were mapped to their respective background sets, and the R software package clusterProfiler (version 4.8.3) was used for enrichment analysis to obtain the results of gene set enrichment. This analysis permitted to obtain a phenotypical point of view of RNA differential expression. For the functional enrichment analysis, the “c5” GO set was used, including three type of sets: molecular function (MF), cellular component (CC) and biological process (BP). TFs-regulated DEGs were analyzed using hTFtarget database^42^.

We analyzed the correlation of BCL6 expression and HCC patients overall survival as well as recurrence survival free as follows: at the Kaplan-Meier Plotter analysis website, (https://kmplot.com/analysis/index.php?p=service&cancer=pancancer_rnaseq), we input gene symbol BCL6, for the cancer type, we chose Liver hepatocellular carcinoma (*n* = 371). Split patients by auto select best cutoff.

### Cell culture

Hepa1-6, H22, Hep53.4, and Hep3B HCC cell lines were purchased from Procell Life Science & Technology (Wuhan, China). Hepa1-6 Hep53.4, and Hep3B cells were routinely cultured in Dulbecco’s modified Eagle medium (DMEM) containing 10% fetal bovine serum (FBS) (Biological Industries, Kibbutz Beit Haemek, Israel) and 1% Penicillin/Streptomycin at 37 °C, 5% CO_2_. H22 cells were cultured in RPMI-1640 containing 10% FBS and 1% Penicillin/Streptomycin at 37 °C, 5% CO_2_. Cell lines were validated by short tandem repeat analysis and tested negative for mycoplasma contamination.

To construct a Bcl6 knockout H22 cell line, CRISPR/Cas9 recognition sites (sgRNA) were designed on exon 1 and exon 3 of Bcl6, respectively. Resulted in deletion of ~4.6 kb of Bcl6 coding DNA, which leads to the failure of Bcl6 protein synthesis. SgRNA (sequence shown below) was cloned into pSpCas9 (BB)-2A-GFP vector, and sgRNA-Cas9 vectors were transfected into 2 × 10^5^ H22 cells via electroporation (Neon™ Transfection System) separately. Transfection condition was 1 600 V and pulse time was 10 ms three times, cells were cultured in fresh culture medium after the electroporation transfection. Two days later, GFP-positive cells were sorted using flow cytometry and seed 1 cell each well in 96-well plates. After 2 weeks culture, two Bcl6 knockout H22 cell line clones were confirmed for knockout efficiency and obtained for further functional study, namely H22 Bcl6 KO#1 and KO#2. Primers for PCR confirmation of genomic Bcl6 deletion were shown below. Briefly, the Bcl6 knockout alleles were amplified by oligo1 and oligo3, and the wild-type alleles were amplified by oligo1 and oligo2. The PCR products were sequenced to confirm the deletion of Bcl6.

SG1: GAAGACAAAATGGCCTCGCCGG

SG2: GGCTCAATAACATCGTTAACAGG

OLIGO1: TGTCAGTGCTTGGCAGAGTAT

OLIGO2: GCTGAGATCTGGAGACAAAG

OLIGO3: CAAACTGAGCCAGAGCTTC

With the same methods, we knocked out Esm1 in H22 wild-type cell line with the following sg RNAs:

SG1: GCTGCTGACCACACTCCtgg

SG2: GCTGCTGACCACACTCCtgg

To confirm the knockout efficiency of ESM1, primers were designed as:

OLIGO1: TGCTGGGTTGAGGAAATTGAT

OLIGO2: ACACCACCTGCATCCAAGCAT

OLIGO3: AGAGCCAGAGTAGAAGATACAG

Esm1 knockout PCR product locates at 570 bp by OLIGO1 (P1) and OLIGO3 (P3), while WT PCR product by OLIGO1 (P1) and OLIGO2 (P2) locates at 600 bp.

In order to construct cell lines that stably overexpress Bcl6 or Esm1, we cloned the CDS sequence into the transposon pB-CMV-MCS-ef1α-puro vector, and introduced the transposon and transposon enzyme into 2 × 10^5^ Hepa1-6 cells or H22 cells by electroporation. The electrotransfection condition was 1200 V, 10 ms pulse time, and the transfection was repeated three times. After electrotransfection, the cells were cultured in fresh culture medium. 2 days later, puromycin (1 μg/ml, Sigma) was added for the selection of overexpression cells.

### Animal studies

C57BL/6N (C57) and BALB/c mice were purchased from Beijing Vital River Laboratory Animal Technology (Beijing, China). For H22 cells, 2 × 10^5^ cells resuspended in 20ul PBS were injected in liver capsule as previously described^43^, and tumor growth were monitored with in vivo imaging. After 2 weeks, mice were sacrificed; liver image and liver weight were recorded. For Hepa1-6, 1 × 10^6^ cells resuspended in 20ul PBS were injected in liver capsule and tumor growth was monitored with in vivo imaging before sacrificed on day 30 post surgery for liver tumor image and liver weight. To deplete CD4^+^T cells and/or CD8^+^T cells, the lytic anti-CD4 (Catalog number: BE0003-1, BioxCell) and anti-CD8 antibodies (Catalog number: BE0061, BioxCell), which were diluted in PBS, were treated via intraperitoneal (i.p.) injection at 100 μg/mouse on days -2,-1,0 and 7 after HCC cells liver orthotopic inoculation.

For the combination therapy of knockout of Bcl6 or Esm1 with anti-PD1 treatment, experiment was designed as shown in Fig. 7k. Briefly, 2 × 10^5^ cells were injected in liver capsule, 7 days later, treat the mice by i.p. injection of anti-PD1 (Catalog number: BE0146, BioxCell) antibody or control IgG (Catalog number: BE0089, BioxCell) (diluted in PBS) with dosage 100 μg/mouse/dose for four doses (two doses 1 week). Sacrificed the mice and analyzed the tumor on day 21.

We anaesthetized the mice with 1–2% isoflurane followed by cervical dislocation as the method of euthanasia according to the ethics. For in vivo liver capsule injection of the HCC cell lines, mice were anaesthetized with 1–2% isoflurane before the surgery. Meloxicam were administrated via subcutaneous injection with dosage of 5 mg/kg mouse weight. Experiment endpoints are 2–3 weeks for the liver growth monitor experiments in H22 Bcl6 knockout and Hepa1-6 Bcl6 overexpressing experiments. For H22 Bcl6 knockout mice survival curve, mouse were sacrificed when the mouse is dying or immobile, or does not respond to gentle stimulation.

We confirm that this study has complied with all relevant ethical regulations for animal testing and research, and the protocol of this study has received ethical approval from the Institutional Animal Care and Use Committee of Chongqing University Cancer Hospital.

### Immunohistochemistry and H&E staining

Immunohistochemically staining was conducted as described previously^43^. Briefly, tissue was formalin-fixed paraffin-embedded before cutting into 4 μm thick sections. Tissue slides were deparaffinized, rehydrated, and blocked with 3% H_2_O_2_ for 15 min. Antigen retrieval was conducted by heating the tissue sections in a pressure cooker for 150 seconds. After cooling to room temperature, sections were incubated with indicated primary antibodies against the following mouse antigens: CD3 (abcam), CD4 (CST), CD8 (abcam) at 4 °C overnight. Then stain the secondary antibody against rabbit and mouse immunoglobulins with the protocol provided by the kit (DAKO, Copenhagen, Denmark). 1–5% 3, 30-diaminobenzidine tetrahydrochloride was used as chromogen for visualization. Sections were counterstained with hematoxylin. For negative control, primary antibody was replaced with phosphate-buffered saline (PBS). Sections were dehydration with 75% EtOH, 90% EtOH, 100% EtOH, and Xylene before mounting with DPX (Avantor).

For frozen sections H&E staining, tissue was fixed in 4% paraformaldehyde at 4 °C overnight before equilibrate in 30% sucrose at 4 °C for 2 days. Then tissue was embedded in O.C.T (Tissue-Tek) and cut into 8 μm sections. For paraffin-embedded tissue H&E staining, paraffin-embedded tissue was deparafinized, rehydrated. Frozen sections and rehydrated paraffin tissue sections were washed with PBS and stained with Hematoxylin (Mayers) for 10 min, then washed with tap water, incubated in PBS for 3 min till slides became blue. Wash with ddH_2_O before staining with eosin Y for 45 s. Wash in ddH_2_O and dehydration before mounting with DPX.

### Western blotting

Cultured cells were lysed with lysis buffer (RIPA lysis supplemented with protease inhibitor and phosphatase inhibitor) and collected by scraper, and dispersed tissue was homogenized on ice for 15 min before centrifugation at 17,000 *g* for 10 min. For HCC patients’ sample, tissue was frozen in liquid nitrogen and then crushed using a mortar and pestle. Homogenized tissue was lysed with lysis buffer described previously. Protein concentration was measured with a BCA protein assay kit (Thermo Fisher). 10 μg protein was loaded and separated on 10–12% SDS-polyacrylamide gel. For cancer cell conditioned medium (CM) collection, Hepa1-6 cells were grown into 80% confluence in 10 cm culture dishes. After three times washing with PBS, the cells were incubated in serum-free medium at 37 °C for 24 h. For H22 CM collection, 1 × 10^6^ H22 cells were seeded in 6 cm dish and cultured in serum-free medium for 24 h. The CM were collected and centrifuged at 1000 g for 5 min followed by filtration with 0.22 μm filters. Secreted proteins in CM were prepared by TCA (Trichloroacetic acid) precipitation. Briefly, 25% v/v TCA was added into 1 ml CM with phosphatase and protease inhibitors, incubated overnight at 4 °C. Then centrifuged at 17,000 *g* for 30 min followed by washing with 1 ml pre-chilled acetone. Centrifuged at 17,000 *g* for 5 min at 4 °C. CM derived secreted protein was lysed with the lysis buffer and separated on 12% SDS-polyacrylamide gel as described previously^44^. Ponceau S staining was used for CM western blot loading control.

After being transferred to Immobilon-NC transfer membrane 0.45 µm, membranes were blocked with 5% skimmed milk at room temperature for 1 h followed by incubation overnight at 4 °C with the indicated primary antibodies: anti-BCL6, anti-Esm1 and anti-GAPDH. Then incubation with the corresponding secondary antibodies, and the signals were developed using SuperSignal Western Blotting Detection Reagent (ThermoFisher, cat#: 34096). Images were acquired using Bio-Rad ChemiDoc Imaging System. All antibodies information was shown in Supplementary Table 3 and the western blotting raw data were shown in Fig. S12. All blots derived from the same experiment were processed in parallel.

### In vivo imaging to monitor tumor growth

Tumor growth in vivo was longitudinally monitored with BLI as previously described^18^. Briefly, mice were anesthetized with 1–2% isoflurane, followed by intraperitoneal injection of 300 mg/kg D-luciferin (APExBIO). Anesthetized mice were placed in a light-tight chamber of the in-vivo imaging system (Perkin Elmer) and photons emitted from live luciferase-expressing cancer cells were captured at 10 min after luciferin injection. BLI signals were expressed in photons per second by drawing a defined ROI at the tumor site.

### Cell proliferation assays

For CCK8 assay, 10,000 H22 cells or Hepa1-6 cells were seeded in 96-well plates (Corning). CCK8 (Bioss, 1:10) was added into each well at the time point of 0, 24, 48, 72 h post cell seeding. The absorbance of A450 was detected with Multimode Microplate Reader (SynergyH1). For in vitro cell growth, 1 × 10^6^ H22 cells were seeded into a 10 cm culture dish, culture at 37 °C, 5% CO_2_ for 48 h, cell number was then counted.

### Flow cytometry

To quantify immune cell infiltrates in liver tumor, mice were sacrificed and liver tumors were collected. Then tumor was dissociated into single cells by mincing into small fragments followed by enzymatic digestion with 1:1 type II collagenase (1000 U/ml in PBS, Worthington) and dispase II (11 U/ml in PBS, Gibco) at 37 °C for 30 min as described previously^45^. Digestion was stopped by adding 10% FBS and the dissociated cells were filtered with 40 μm strainer to obtain single cells. For surface staining, cells were then blocked with 2% normal rabbit serum followed by staining with fluorochrome-conjugated antibodies against the following mouse antigens: CD45, CD3, CD4, CD8, PD-1, Tim3, Ly6G, Ly6C, CD11b at 4 °C for 30 min, then washed with 2% FBS-containing PBS. Detailed antibody information please see Supplementary Table 3. For intracellular staining of Th differentiation markers Ifnγ, Tnfα, Il-4 and Il17, liver tumor-derived single cells were re-stimulated in vitro with phorbol-12-myristate-13-acetate (100 ng/ml, Sigma) and Brefeldin A (BD Biosciences) for 6 h at 37 °C,5% CO_2_ in RPMI 1640 medium containing 10% FBS and 1% penicillin/streptomycin. Cells were firstly blocked and stained with surface markers including CD45, CD3, CD4 and CD8, then cells were fixed and permeablized following the protocol provided by the intracellular staining kit (eBiosciences). For T cells proliferation marker Ki67 staining, dissociated single cells were fixed and permeablized following the protocol provided by the kit and stained with rabbit anti-Ki67 primary antibody (abcam) in 4 °C for 30 min, washed three times, then stained with anti-rabbit secondary antibodies conjugated with Alexa fluor 647 at 4 °C for 30 min, primary staining of rabbit IgG was used as control. Cells were washed three times before analysis on a flow cytometer (Beckman CytoflexLX) and FACS data were analyzed with FlowJo software (Tree star).

For mass cytometry, H22 cells were transplanted in liver capsule for 2 weeks before sacrificed for tumor collection. Then tumor single cells were harvested by enzymatic digestion as mentioned above. Antibodies used for immune cells are from FLUIDIGM panel kit (Catolog no. 201306). The clone number for each antibody is as follows, Ly-6G/Gr-1(RB6-8C5), CD11c(N418), CD69 (H1.2F3), CD45(30-F11), CD11b/Mac-1 (M1/70), CD19(6D5), CD25 (3C7), CD3e (145-2C11), TER-119(TER-119), CD62L (MEL-14), CD8a (53-6.7), TCRβ (H57-597), NK1.1 (PK136), CD44 (IM7), CD4 (RM4-5), B220 (RA3-6B2).

### Single-cell RNA sequencing analysis

H22 were injected orthotopically in liver capsule. At 2 weeks post-surgery, total tumor single cell suspension were obtained using enzymatic digestion as described previously. Cell viability was assessed by trypan blue staining (cell viability > 90%). Samples were prepared using a 10x Genomics Single Cell 3’ v3 Reagent Kit according to the manufacturer’s instructions. Single Cell 3’ v3 Reagent Kit according to the manufacturer’s instructions. Single-cell libraries were prepared and sequenced on an DNBSEQ T7 (BGI). Raw data were imported to CellRanger (version 2.2.0) and aligned to mouse reference genome (GRCm38). In total, 32,285 genes were detected for each group, and 13078, 30533 cells were captured for Bcl6 knockout (KO) and control wildtype (WT) group, respectively. Cells with <200 total expressed genes, >25% mitochondria-expressed genes, or with >20% ribosomal genes were removed. Genes detected in fewer than three cells, mitochondrial or ribosomal genes were removed since their expression is very variable. DoubletFinder (version 2.0.3) was employed to remove doublets. After the quality control, total of 21,424 genes were retained, and 10292, 29,454 cells were obtained for KO and WT group, respectively. Due to the varied cell number between KO and WT group, we selected 10,000 cells per group randomly for further analysis. Harmony package (version 0.1.0) was then applied to remove the batch effect. Following normalization, 2000 highly variable genes (identified by FindVariableGenes function of Seurat package) were used as input to principal component analysis. The first 30 principal components (PCs) were estimated by an Elbow plots. Then, these principal components were used for calculating Uniform manifold approximation and projection (UMAP) embedding and cell clusters were identified using the FindClusters function based on their PCs (resolution = 0.5). Cell clusters were annotated according to the marker genes composition. For T cells, total T cells were selected and re-clustered using Findcluster function with resolution 0.5, and annotated according to T cell subpopulation marker genes.

### ICAM-1 T cell adhesion assay

For CD4^+^T cells isolation, mouse spleen-derived single cells were stained with anti-CD4 PE antibody (Biolegend) at 4 °C for 30 min, then washed with PBS containing 2% FBS and stained with anti-PE magnetic beads with the indicated volume (Cat#:130-048-801, Miltenyi Biotec). CD4^+^T cells were then positively sorted with LS Columns and MidiMACS^TM^ separator (Cat#:130-042-401, Miltenyi Biotec). T cells CAM-1/LFA-1-I mediated adhesion assay was conducted as described previously^46^. Briefly, CD4^+^T cells sorted from mouse spleen were stained with CFSE at 37 °C for 8 min at room temperature, then washed and stimulated with: adhesion buffer alone as unstimulated control (PBS + 1 mM CaCl_2_ + 2 mM MgCl_2_ + 0.5% bovine serum albumin), PMA(10 ng/ml in adhesion buffer), PMA (10 ng/ml)+H22 WT CM (adhesion buffer: CM volume ratio 1:1), PMA(10 ng/ml)+H22 Bcl6 KO#1 CM, PMA (10 ng/ml)+H22 Bcl6 KO#2 CM (adhesion buffer: CM volume ratio 1:1), PMA (10 ng/ml)+H22 Bcl6 KO Ctrl (vector) CM (adhesion buffer: CM volume ratio 1:1), PMA(10 ng/ml)+H22 Bcl6 KO#1 Esm1 CM (adhesion buffer: CM volume ratio 1:1), or with mouse Esm1 recombinant protein(300 ng/ml, Sino Biological, cat# 51108). 96 well plate was pre-coated with coating buffer (ICAM-1 10 μg/ml in PBS + 1 mM CaCl_2_, 2 nM MgCl_2_) at 37 °C for 1 h. Then aliquot 50 μl (1 × 10^5^ cells) of the simulated T cells or control unstimulated cells into the coated wells (3 wells per group), and aliquot PMA treated T cells in three uncoated wells as control. Centrifuge the plate at 100 *g* for 5 min at room temperature, then incubate the plate in 37 °C incubator for 30 min. Wash away non-adherent T cells by adding 150 μl of warm adhesion buffer into each well, gently shake for few seconds before removing the medium from the wells. Repeat the washing step three times. Take the picture of the adherent GFP^+^T cells and calculate the percentage of the adherent cells.

### Statistical analysis

Statistical analyses were performed with Graphpad prism 9.0. Data were demonstrated as arithmetic mean ± S.E.M unless otherwise mentioned. Statistical analysis was performed using the unpaired student’s *t* test for data from two groups; while data from more than two groups was compared using one-way ANOVA. Kaplan-Meier’s survival curves and log-rank tests were applied to compare the survival between groups. Significance was accepted when statistic *p* value < 0.05.

### Reporting summary

Further information on research design is available in the Nature Research Reporting Summary linked to this article.

### Supplementary information

Supplementary Information

Reporting summary

## Data Availability

All relevant data are available on request from the corresponding authors (Cheng Qian, email: cqian3184@163.com Juanjuan Shan, email: juanjuansh@gmail.com). The single-cell RNA sequencing raw data for Bcl6 knockout and control H22-derived tumors has been deposited in Sequence Read Archive (SRA) dataset (Accession code: PRJNA1092723). RNA sequencing raw data for H22 wild-type cell line and Bcl6 knockout cell line has been deposited in Sequence Read Archive (SRA) data set (Accession code: PRJNA1092336).

## Code availability

No custom code or scripts were used in this study. For the analysis of transcriptional factors involved in T cell infiltration from TCGA data, we used TCGA biolinks package version 2.30.0, DESeq2 package version 1.42.1, org.Hseg.db version 3.18.0, ClusterProfiler package version 4.8.3, enrichplot package version 1.22.0, ggplot2 package version 3.5.0, R package version 4.3.1, Homer version 4.10. For single cell RNA sequencing data analysis, we used CellRanger version 2.2.0, DoubletFinder version 2.0.3, Harmony package version 0.1.0. Specific parameters used in bioinformatics analysis were shown in the Methods.
