# Supplementary material for: B cell lymphoma 6 promotes hepatocellular carcinoma progression by inhibiting tumor infiltrating CD4+T cell cytotoxicity through ESM1
Source: NPJ Precis Oncol. 2024 Jul 1;8:139. doi: 10.1038/s41698-024-00625-7 (PMC11220024; doi:10.1038/s41698-024-00625-7)
Supplement: Supplementary file 1 — Supplementary Information [file 41698_2024_625_MOESM1_ESM.pdf]

Supplementary Figures

Fig. S1

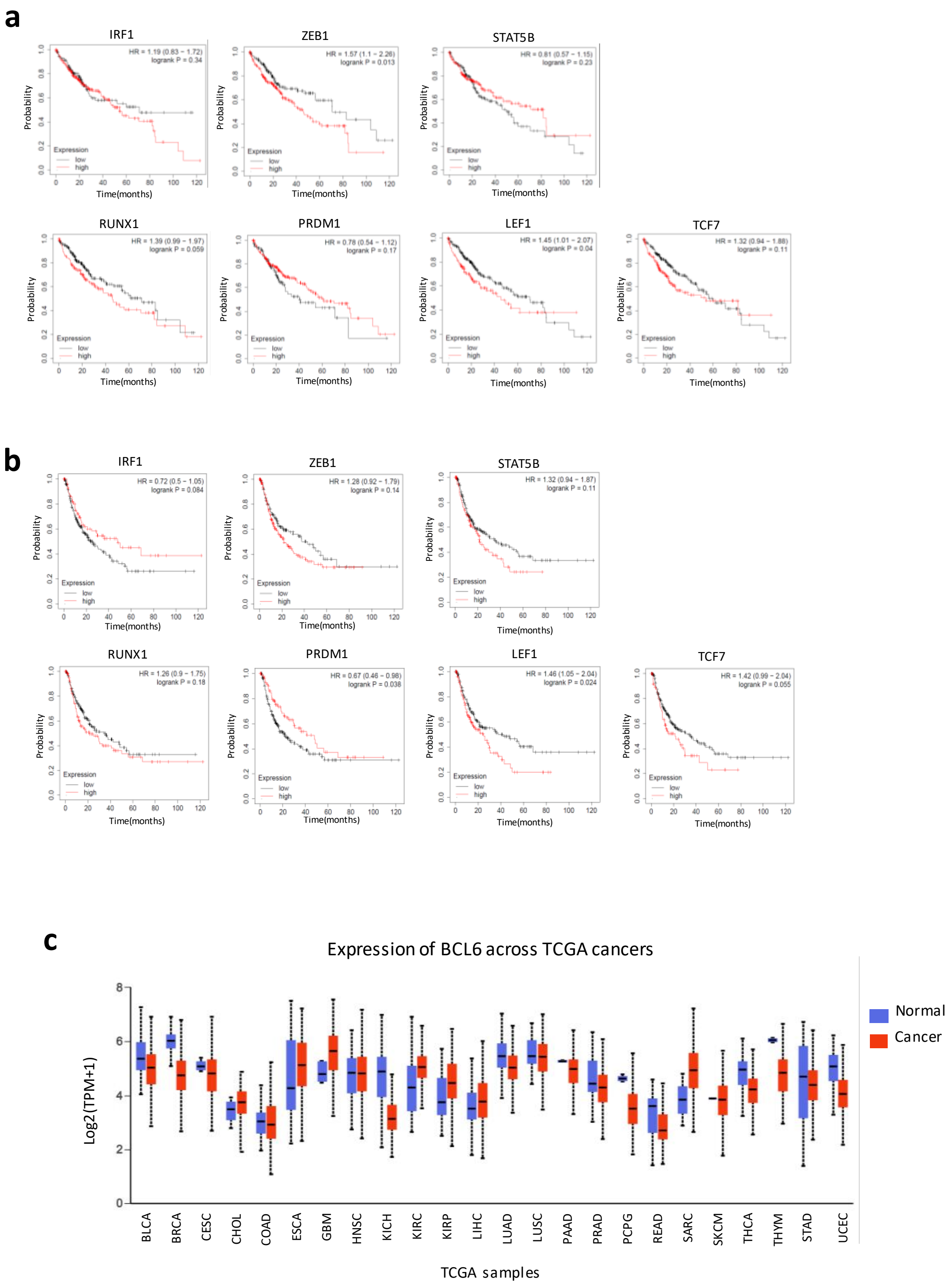

Fig. S1. Overall survival (OS) and recurrence free survival (RFS) for the transcriptional factors. (a) Overall survival for the TFs. (b) Recurrence free survival for the TFs. (c) Pan-cancer view of BCL6 expression using data from TCGA.

Fig. S2

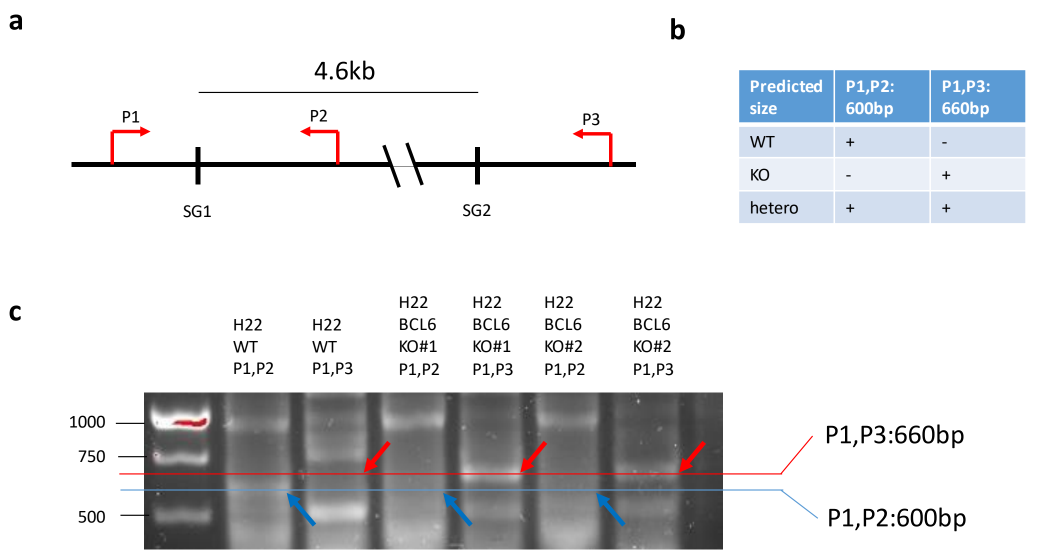

Fig. S2. PCR for Bcl6 knockout confirmation. (a) Scheme for Bcl6 knockout primer design and the predicted size for the PCR product (b). (c) PCR confirmation of Bcl6 knockout using H22 cell genome DNA.

Fig. S3

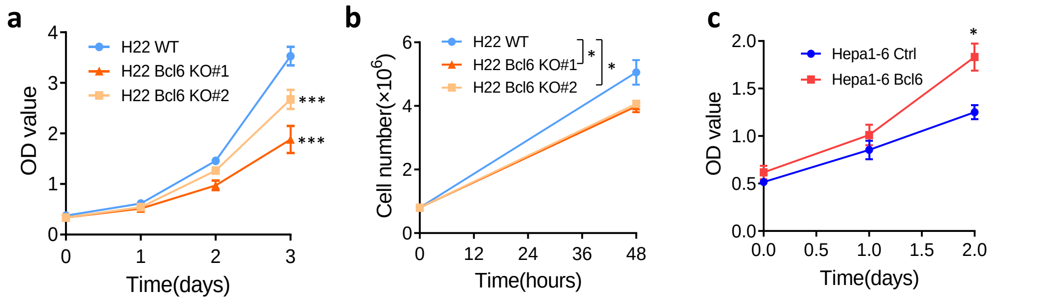

Fig. S3. Bcl6 promotes HCC cancer cell growth in vitro. CCK8 assay (a) and cell growth curve (b) for H22 wild type and Bcl6 knockout group, n=3 each group. (c) CCK8 assay for Hepa1-6 ctrl and Bcl6 overexpression cell lines, n=3 each group. *p<0.05, ***p<0.001.

Fig. S4

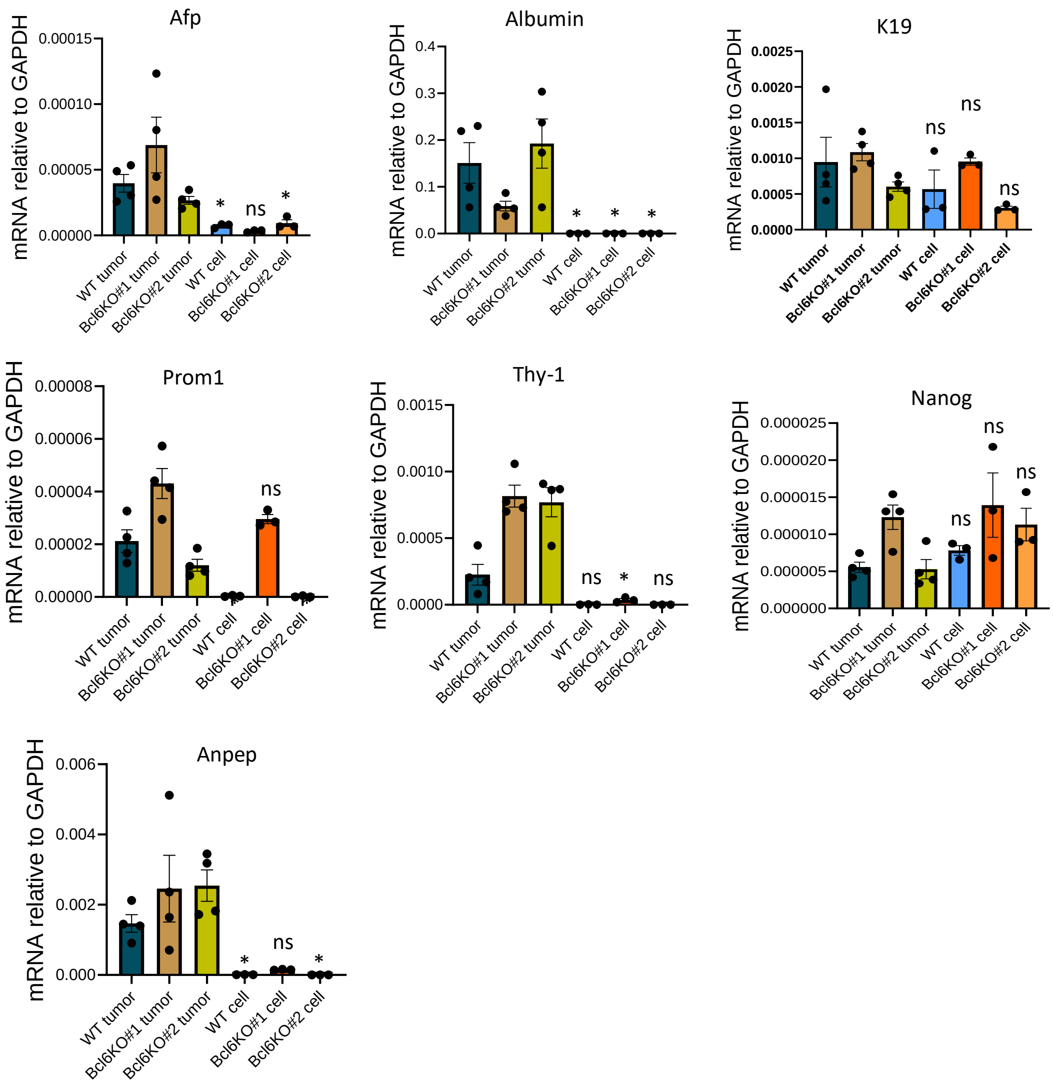

Fig. S4. H22 tumor expression higher differentiation and stem cell genes than H22 cell line. qPCR data for differentiation marker genes Atp, Albumin, K19 and cancer stem cell marker genes Prom-1, Thy-1, Nanog, Anpep in H22 tumor and H22 cell lines with and without knockout of Bcl6. n=4 per group, **p*<0.05, ***p*<0.01, ****p*<0.001, ns: no significance.

Fig. S5

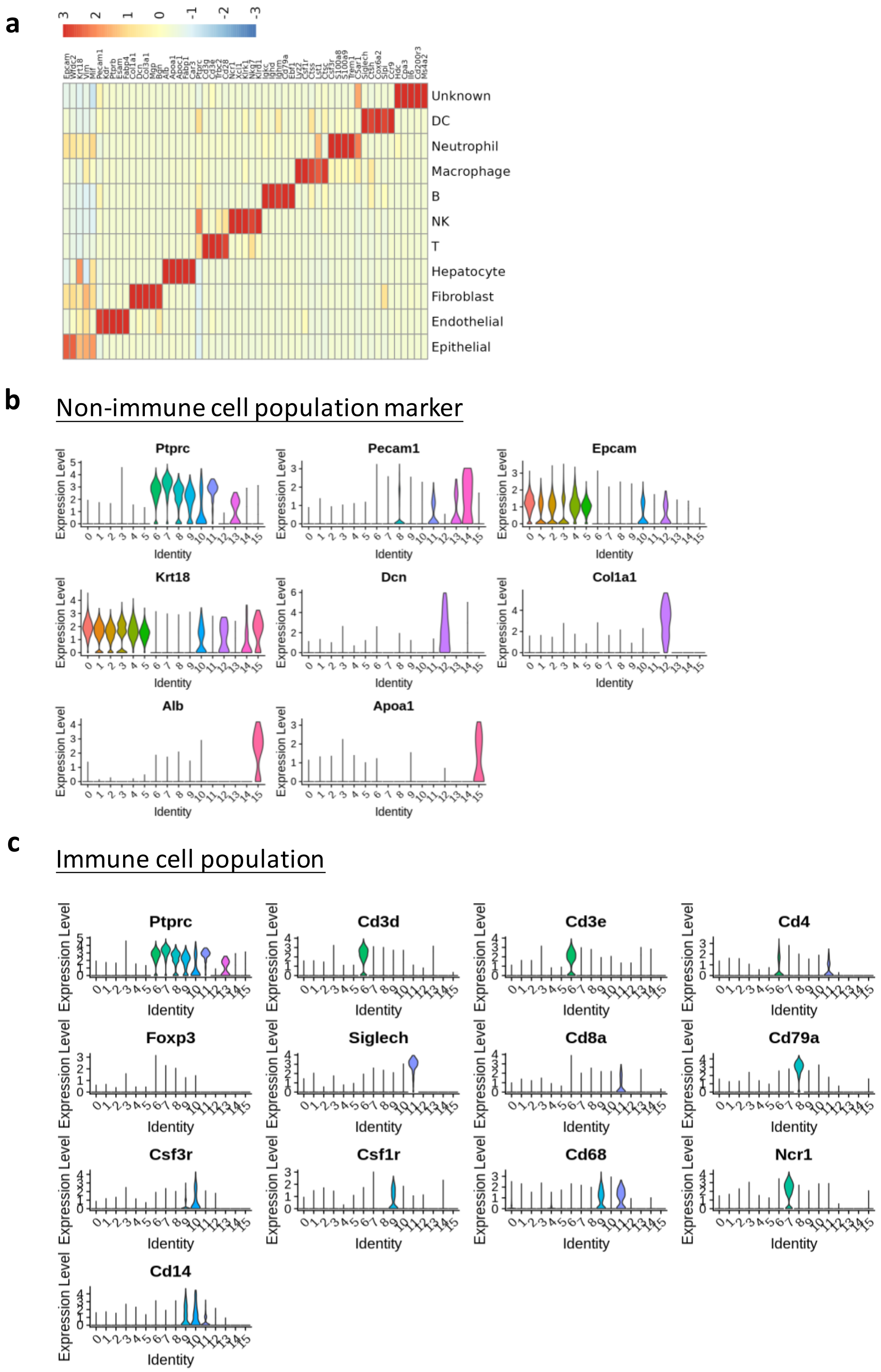

Fig. S5. Markers used for single cell RNA sequencing cell cluster definition.(a) Heatmap showing immune cell as well as non-immune cell gene expression patterns.(b) Expression of representative marker genes used for cell cluster definition in each cell cluster for non-immune cells (b) and immune cells (c), cell cluster definition: 0:

Epithelial_c1, 1: Epithelial_c2, 2: Epithelial_c3, 3: Epithelial_c4, 4: Epithelial_c5, 5: Epithelial_c6, 6: CD4+T, 7: NK, 8: B, 9: Macrophage, 10: Neutrophil, 11: DC, 12: Fibroblast, 13: Unknown, 14: Endothelial, 15: Hepatocyte.

Fig. S6

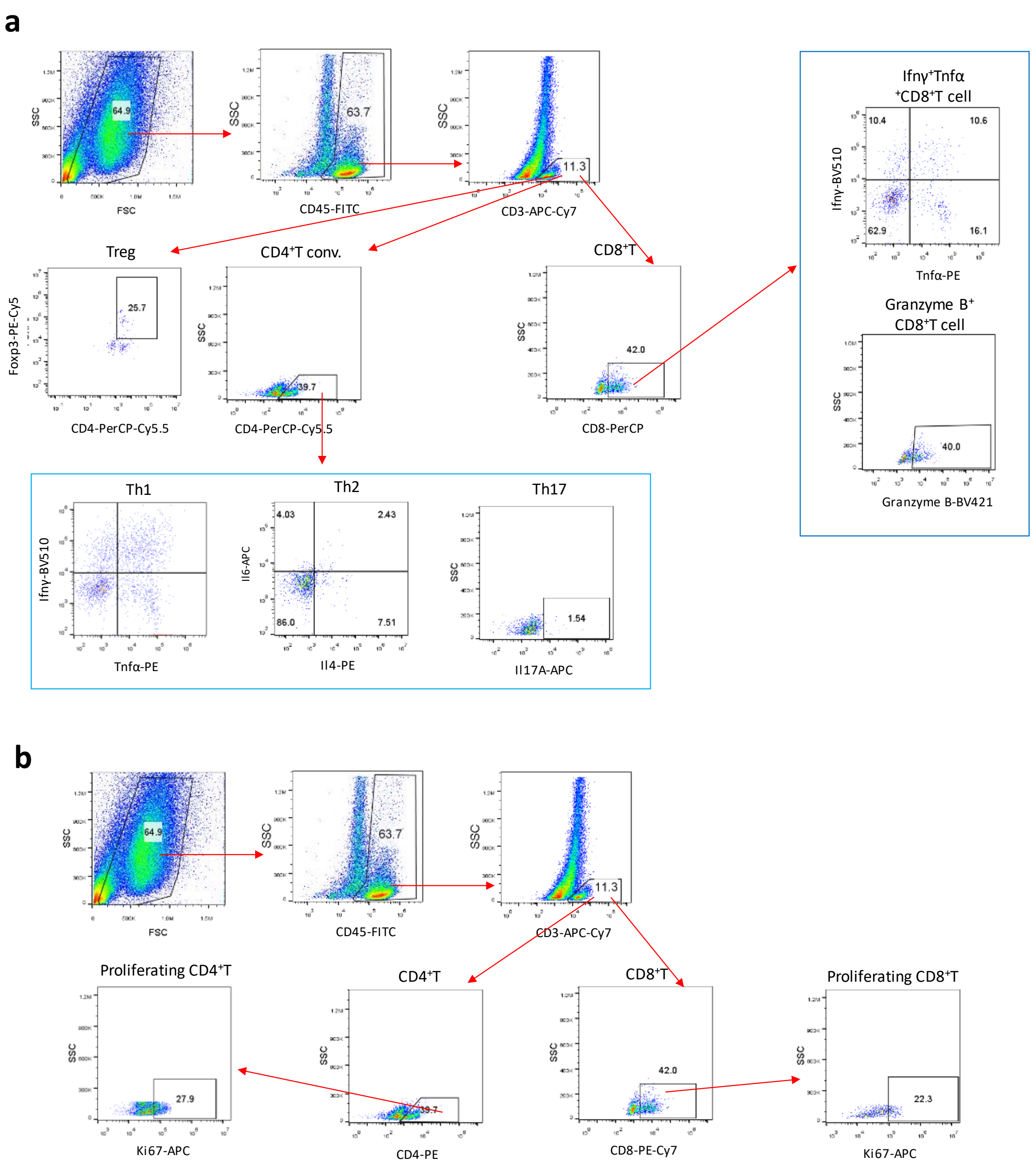

Fig. S6. Gating strategy for CD4$^+$T cells and CD8$^+$T cells. (a) Gating strategy for CD45$^+$immune cells, CD3$^+$T cells, Th, Treg and CD8$^+$T cells infiltration and differentiation presented on Fig. 4a, 4b, 4d, 4e, 4f, 4h-4l. (b) Gating strategy for proliferating T cells presented on Fig. 4g.

Fig. S7

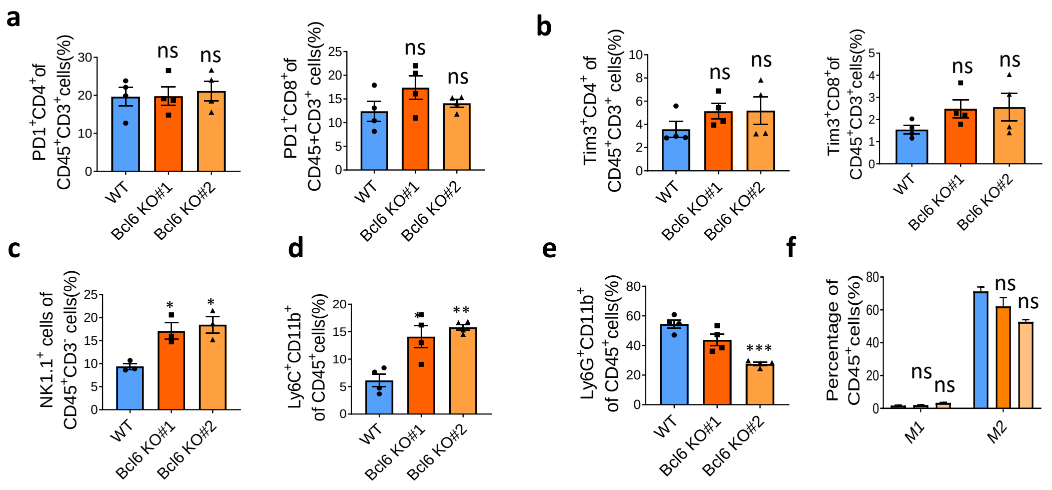

Fig. S7. Effect of Bcl6 knockout on T cell exhaustion and innate immune cell infiltration. (a) Flow cytometry data indicated that Bcl6 knockout does not affect the percentage of PD1$^+$CD4$^+$ T cells or PD1$^+$CD8$^+$T cells=4 each group.(b) Tim3$^+$T cells infiltration in tumor was also not affect by Bcl6 knockout=4 each group. Flow cytometry data showed that knockout of Bcl6 increased NK cells (c) and Ly6C high MDSC infiltration (d), but decreased Ly6G high MDSC infiltration (e), no effect on M1 or M2 polarization (f), n=3-4 per group. **p*<0.05, ***p*<0.01, ****p*<0.001.

Fig. S8

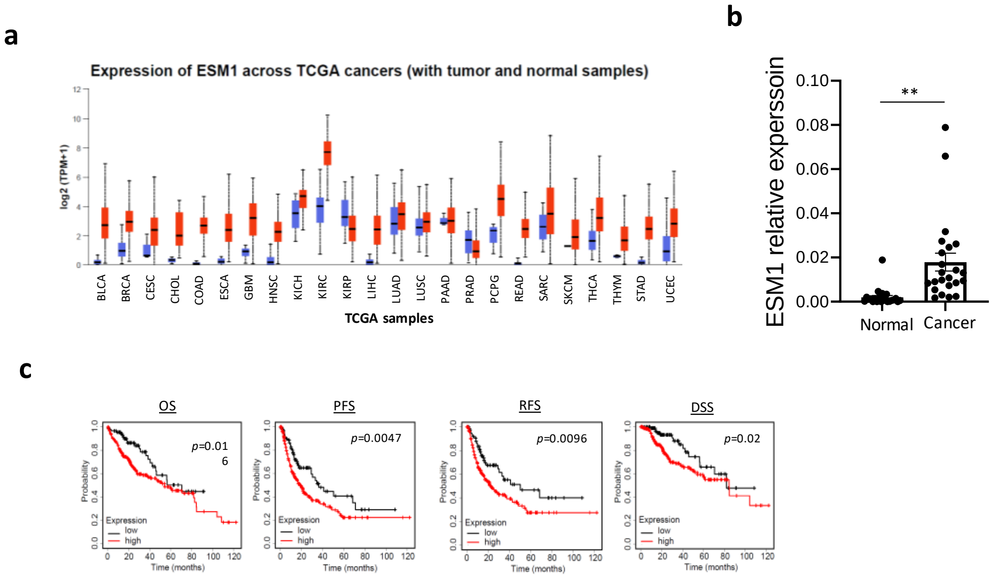

Fig. S8. ESM1 expression in pan cancer data as well as function on liver cancer cell. (a) Pan-cancer view of ESM1 expression. (b)ESM1 expression using qPCR analysis. (c) ESM1 on liver cancer survival based on TCGA data. OS**:** overall survival, PFS: progression-free survival, RFS: recurrence-free survival, DSS: disease free survival (DSS). ***p*<0.01.

Fig. S9

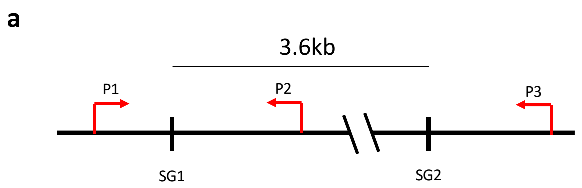

b

Predicted size	P1,P2: 600bp	P1,P3: 570bp
WT	+	-
KO	-	+
hetero	+	+

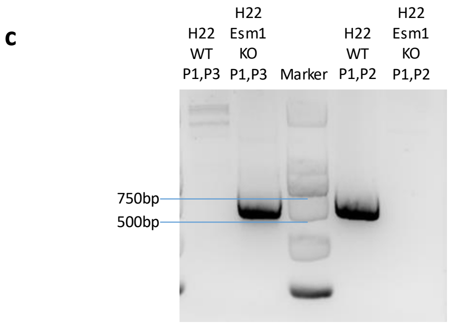

Fig. S9. PCR for Esm1 knockout confirmation. (a) Scheme for Esm1 knockout primer design and the predicted size for the PCR product (b). (c) PCR confirmation of Esm1 knockout using H22 cell genome DNA.

Fig. S10

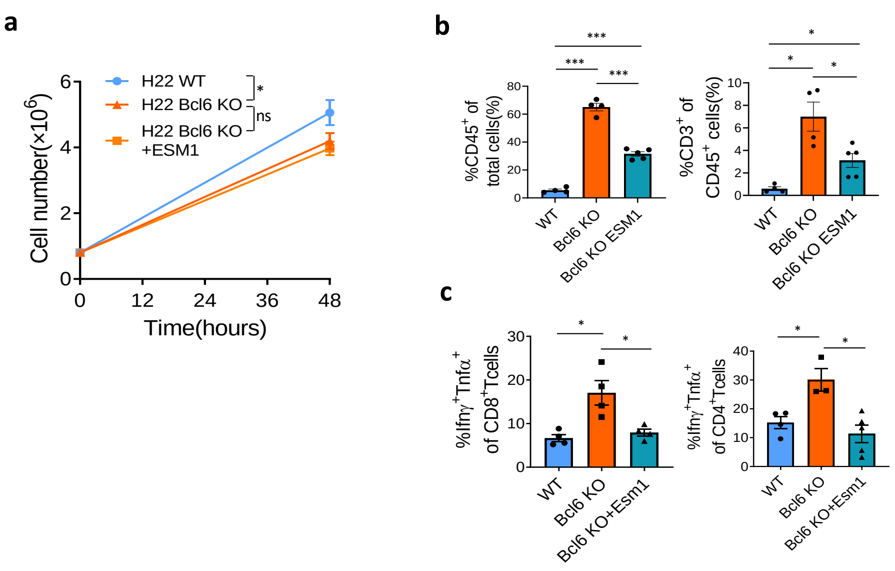

Fig. S10. ESM1 expression in pan cancer data as well as function on liver cancer cell. (a) H22 wild type, Bcl6 knockout and Bcl6 knockout with Esm1 overexpression cells proliferation were monitored within 48 hours and showed that Esm1 does not affect cancer cell proliferation, n=3 each group. Flow cytometry quantification for tumor infiltration immune cells (b) and T cells activation (c) for H22 WT, Bcl6 KO and Bcl6 KO+Esm1 overexpression at 2 weeks post H22 injection, n=4-5 per group. **p*<0.05, ****p*<0.001, ns: no significance.

Fig. S11

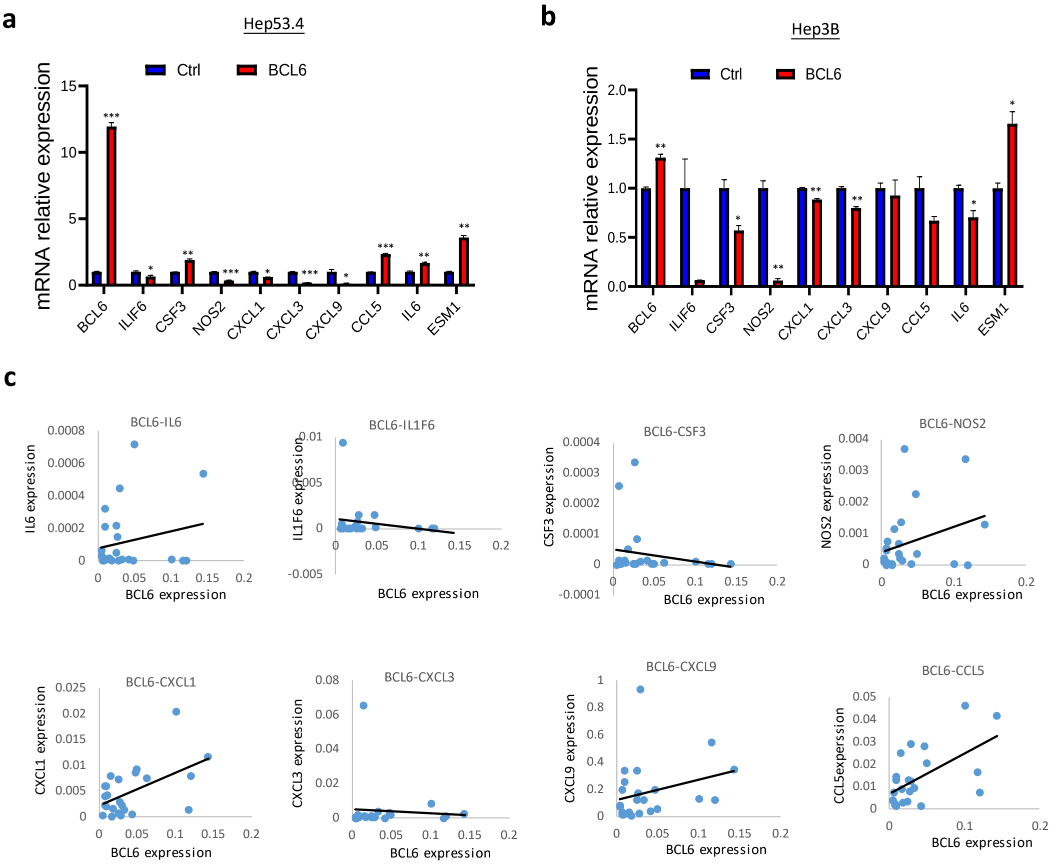

d

Pearson correlation value between BCL6 and inflammatory related genes									
	IL6	IL1F6	CSF3	CXCL1	CXCL3	CXCL9	CCL5	NOS2	ESM1
Male	0.29	-0.22	-0.18	0.61	0.23	0.56	0.66	0.26	0.61
Femal	-0.12	-0.18	-0.21	0.76	-0.06	0.03	0.73	0.12	-0.06
Total	0.21	-0.18	-0.18	0.52	-0.08	0.27	0.58	0.30	0.51

Fig. S11. BCL6 and inflammatory genes in different cell lines and HCC patient samples. qPCR for inflammatory genes and ESM1 in Hep53.4 (a) and Hep3B (b)with or without BCL6 overexpression. n=3 per group. (c) Correlation of BCL6 expression with inflammatory genes and ESM1 in HCC patients samples based on qPCR data. (d) Pearson correlation value for BCL6 and inflammatory genes and ESM1 in total HCC samples and sex separated groups. *p<0.05, **p<0.01, ***p<0.001, ns: no significance.

Fig. S12

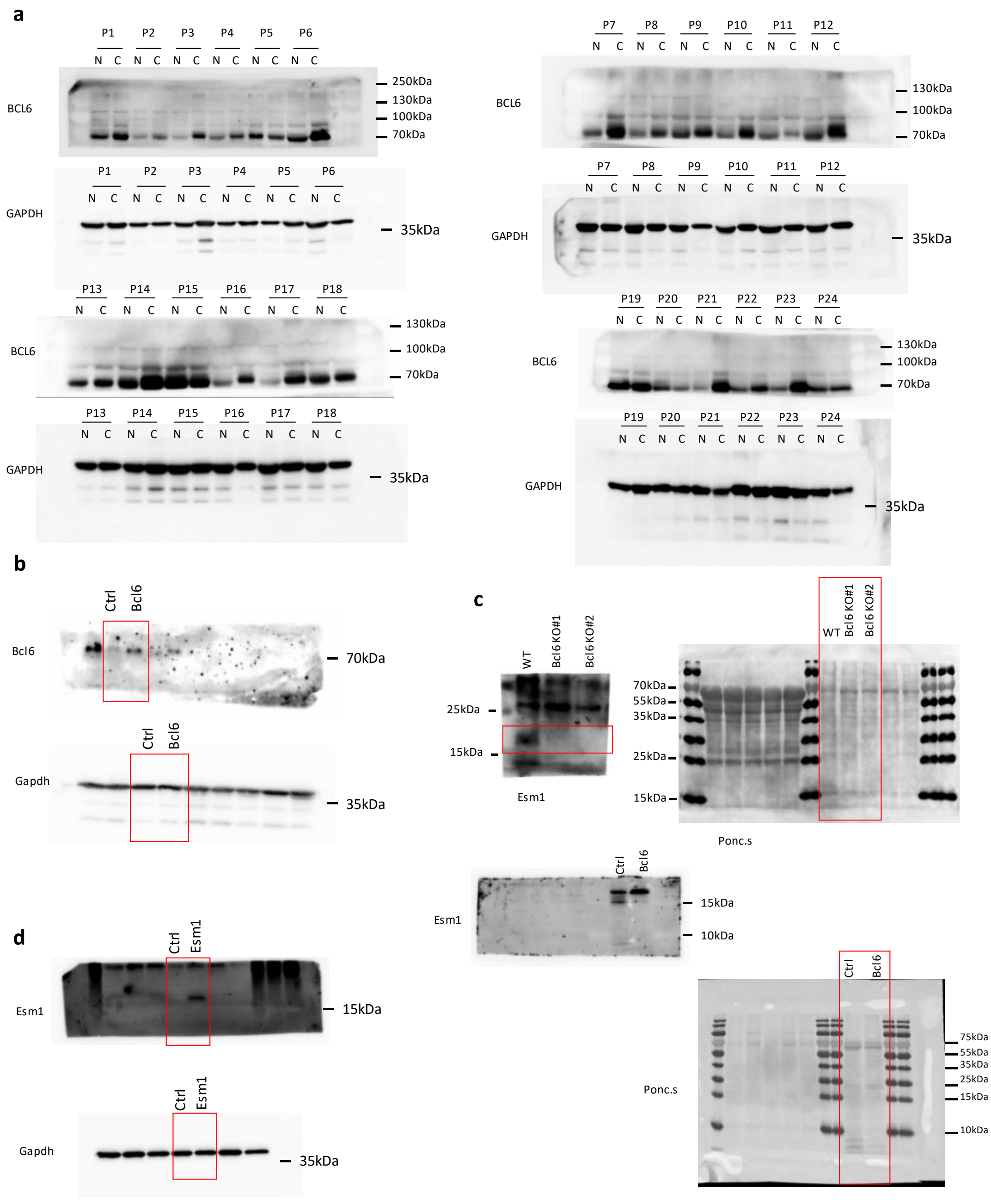

Fig. S12. Western blotting raw data. (a) Uncropped scans of the western blotting for detecting of BCL6 expression in human patient samples as shown in Fig. 1h. (b) Uncropped scans of the western blotting for Bcl6 overexpression in Hepa1-6 overexpression as shown in Fig. 2i. (c) Uncropped scans of the western blotting for Esm1 expression in the medium of H22 WT or Bcl6 knockout cell lines, and Hepa 1-6 ctrl and Bcl6 overexpression cell lines as shown in Fig. 6g. (d) Western blotting scans for Esm1 expression in H22 Bcl6 KO control line and Bcl6 KO+Esm1 overexpression line.

Supplementary Tables

Supplementary Table 1. Patient information for HCC paraffin samples

Tissue type(Cancer:C, adjacent normal tissue:N)	Surgery data(year-month)	Living state	Survival time(month)	Sex	Age	Stage(I-IV)
C and N	2007-1	Live	80	Male	unknown	III
C and N	2007-2	Live	79	Male	67	II
C and N	2007-3	Live	78	Male	58	II
C and N	2007-4	Live	77	Male	62	I - II
C and N	2007-8	Live	73	Male	50	II
C and N	2008-2	Live	67	Male	43	II
C and N	2008-3	Live	66	Male	52	I - II
C and N	2008-7	Live	62	Male	61	II
C and N	2008-12	Live	57	Male	54	II
C and N	2008-12	Live	57	Male	48	I
C and N	2008-12	Live	57	Male	48	II
C and N	2008-12	Live	57	Male	70	II
C and N	2008-12	Live	57	Male	70	II
C and N	2009-2	Live	55	Male	52	II - III
C and N	2009-2	Live	55	Male	50	II
C and N	2009-2	Live	55	Male	55	II
C and N	2009-3	Live	54	Male	54	II
C and N	2009-3	Live	54	Male	34	II
C and N	2009-3	Live	54	Male	50	II
C and N	2009-6	Live	51	Male	49	II
C and N	2009-6	Live	51	Male	55	III
C and N	2009-7	Live	50	Male	40	II - III
C and N	2009-7	Live	50	Male	59	II - III
C and N	2009-7	Live	50	Male	63	II

C and N	2009-8	Live	49	Male	63	Ⅰ-Ⅲ
C and N	2009-10	Live	47	Male	73	Ⅱ
C and N	2009-11	Live	46	Male	47	Ⅱ
C	2007-4	Live	77	Male	40	Ⅱ-Ⅲ
C	2007-7	Live	74	Male	58	Ⅰ-Ⅲ
C and N	2007-8	Live	73	Female	44	Ⅱ
C and N	2007-8	Live	73	Female	58	Ⅱ
C and N	2008-12	Live	57	Female	65	Ⅱ
C and N	2009-3	Live	54	Female	67	Ⅱ
C and N	2009-11	Live	46	Female	70	Ⅱ-Ⅲ
C and N	2007-2	Death	11	Male	65	Ⅲ
C and N	2007-4	Death	11	Male	49	III
C and N	2007-4	Death	14	Male	71	Ⅱ
C and N	2007-4	Death	13	Male	70	Ⅱ
C and N	2007-4	Death	49	Male	55	Ⅱ
C and N	2007-6	Death	4	Male	49	Ⅲ
C and N	2007-7	Death	43	Male	69	Ⅱ-Ⅲ
C and N	2007-8	Death	29	Male	51	Ⅱ-Ⅲ
C and N	2007-9	Death	69	Male	52	Ⅱ
C and N	2008-1	Death	29	Male	57	Ⅲ
C and N	2008-3	Death	4	Male	56	Ⅲ
C and N	2008-3	Death	16	Male	46	Ⅲ
C and N	2008-3	Death	4	Male	53	Ⅱ
C and N	2008-3	Death	8	Male	48	Ⅱ
C and N	2008-3	Death	17	Male	54	Ⅱ
C and N	2008-4	Death	11	Male	57	Ⅱ-Ⅲ
C and N	2008-4	Death	14	Male	56	Ⅰ
C and N	2008-6	Death	4	Male	43	Ⅱ
C and N	2008-8	Death	5	Male	49	Ⅱ-Ⅲ
C and N	2008-8	Death	1	Male	68	Ⅲ
C and N	2008-11	Death	3	Male	42	Ⅱ-Ⅲ
C and N	2008-12	Death	57	Male	42	Ⅱ
C and N	2008-12	Death	17	Male	39	Ⅱ-Ⅲ
C and N	2008-12	Death	17	Male	51	Ⅱ
C and N	2008-12	Death	7	Male	38	Ⅱ
C and N	2009-1	Death	6	Male	37	Ⅱ
C and N	2009-1	Death	15	Male	53	Ⅱ-Ⅲ
C and N	2009-1	Death	30	Male	52	Ⅱ
C and N	2009-1	Death	34	Male	67	Ⅱ-Ⅲ
C and N	2009-2	Death	32	Male	57	Ⅱ-Ⅲ
C and N	2009-3	Death	5	Male	51	Ⅱ
C and N	2009-3	Death	4	Male	65	Ⅱ
C and N	2009-3	Death	6	Male	54	Ⅱ
C and N	2009-5	Death	28	Male	63	Ⅱ

C and N	2009-5	Death	33	Male	45	Ⅱ-Ⅲ
C and N	2009-6	Death	27	Male	53	Ⅱ-Ⅲ
C and N	2009-7	Death	12	Male	49	Ⅲ
C and N	2009-7	Death	16	Male	25	Ⅱ-Ⅲ
C and N	2009-7	Death	5	Male	40	Ⅱ
C and N	2009-7	Death	9	Male	58	Ⅰ-Ⅲ
C and N	2009-8	Death	25	Male	26	Ⅰ-Ⅲ
C and N	2009-9	Death	2	Male	58	Ⅱ-Ⅲ
C and N	2009-10	Death	7	Male	69	Ⅱ
C and N	2009-10	Death	5	Male	56	Ⅱ
C and N	2009-11	Death	30	Male	62	Ⅰ-Ⅱ
C and N	2009-11	Death	19	Male	57	Ⅱ-Ⅲ
C and N	2009-11	Death	10	Male	59	Ⅱ-Ⅲ
C and N	2009-11	Death	16	Male	38	Ⅱ
C and N	2009-11	Death	35	Male	65	Ⅱ
C and N	2009-11	Death	12	Male	54	Ⅱ
C	2007-8	Death	32	Male	56	Ⅱ
C	2008-2	Death	35	Male	52	Ⅰ-Ⅱ
C	2008-11	Death	43	Male	61	Ⅱ-Ⅲ
C	2009-5	Death	4	Male	56	Ⅱ-Ⅲ
C	2009-11	Death	2	Male	44	Ⅱ
C and N	2008-2	Death	25	Female	48	Ⅱ
C and N	2008-8	Death	29	Female	66	Ⅱ
C and N	2009-3	Death	16	Female	54	Ⅱ
C and N	2009-7	Death	10	Female	44	Ⅱ
C and N	2007-8	Death	2	Female	57	Ⅱ-Ⅲ

Supplementary Table 2. Patient information for HCC fresh samples

Sex	Date of birth	Age at surgery	Tissue type(Cancer tissue:C, adjacent normal tissue:N)	Cancer type	Tissue number
Female	1971-07-18	52	C	Hepatocellular carcinoma	1 C
Female	1971-07-18	52	N	Hepatocellular carcinoma	1 N
Female	1952-05-04	70	C	Hepatocellular carcinoma	2 C
Female	1952-05-04	70	N	Hepatocellular carcinoma	2 N
Female	1967-04-16	53	C	Hepatocellular	3 C

				carcinoma	
Female	1967-04-16	53	N	Hepatocellular carcinoma	3 N
Female	1965-03-08	55	C	Hepatocellular carcinoma	4 C
Female	1965-03-08	55	N	Hepatocellular carcinoma	4 N
Female	1993-12-09	30	C	Hepatocellular carcinoma	5 C
Female	1993-12-09	30	N	Hepatocellular carcinoma	5 N
Female	1981-02-16	38	C	Hepatocellular carcinoma	6 C
Female	1981-02-16	38	N	Hepatocellular carcinoma	6 N
Female	1949-12-30	71	C	Hepatocellular carcinoma	7 C
Female	1949-12-30	71	N	Hepatocellular carcinoma	7 N
Male	1956-08-09	65	C	Hepatocellular carcinoma	8 C
Male	1956-08-09	65	N	Hepatocellular carcinoma	8 N
Male	1971-11-05	52	C	Hepatocellular carcinoma	9 C
Male	1971-11-05	52	N	Hepatocellular carcinoma	9 N
Male	1944-11-26	78	C	Hepatocellular carcinoma	10 C
Male	1944-11-26	78	N	Hepatocellular carcinoma	10 N
Male	1964-09-12	59	C	Hepatocellular carcinoma	11 C
Male	1964-09-12	59	N	Hepatocellular carcinoma	11 N
Female	1965-01-15	56	C	Hepatocellular carcinoma	12 C
Female	1965-01-15	56	N	Hepatocellular carcinoma	12 N
Female	1971-10-27	51	C	Hepatocellular carcinoma	13 C
Female	1971-10-27	51	N	Hepatocellular carcinoma	13 N

Male	1959-07-05	64	C	Hepatocellular carcinoma	14 C
Male	1959-07-05	64	N	Hepatocellular carcinoma	14 N
Male	1973-07-27	49	C	Hepatocellular carcinoma	15 C
Male	1973-07-27	49	N	Hepatocellular carcinoma	15 N
Male	1973-07-20	50	C	Hepatocellular carcinoma	16 C
Male	1973-07-20	50	N	Hepatocellular carcinoma	16 N
Male	1964-06-17	58	C	Hepatocellular carcinoma	17 C
Male	1964-06-17	58	N	Hepatocellular carcinoma	17 N
Male	1976-04-09	46	C	Hepatocellular carcinoma	18 C
Male	1976-04-09	46	N	Hepatocellular carcinoma	18 N
Male	1967-02-14	56	C	Hepatocellular carcinoma	19 C
Male	1967-02-14	56	N	Hepatocellular carcinoma	19 N
Male	1962-06-07	59	C	Hepatocellular carcinoma	20 C
Male	1962-06-07	59	N	Hepatocellular carcinoma	20 N
Male	1959-12-25	64	C	Hepatocellular carcinoma	21 C
Male	1959-12-25	64	N	Hepatocellular carcinoma	21 N
Female	1953-06-12	70	C	Hepatocellular carcinoma	22 C
Female	1969-08-15	51	N	Hepatocellular carcinoma	22 N
Female	1965-01-15	56	C	Hepatocellular carcinoma	23 C
Female	1971-10-27	51	C	Hepatocellular carcinoma	23 N
Female	1964-12-28	59	C	Hepatocellular carcinoma	24 C
Male	1964-09-12	59	C	Hepatocellular carcinoma	24 N

Supplementary Table 3. Antibodies used in this study

Protein targets	Assays	Catalog	Supplier	Clone name	Lot number
BCL6	WB/IHC	ab203619	abcam	Polyclonal	GR3252536-4
FLAG	WB/ChIP	14793S	Cell Signaling Technology	D6W5B	5
ESM1	WB/Flow cytometry	ab103590	abcam	Polyclonal	1019528-1
GAPDH	WB	2118S	Cell Signaling Technology	14C10	14
CD3	WB/IHC	ab5690	abcam	Polyclonal	GR3410787-1
CD4	IHC	ab183685	abcam	EPR19514	GR3375645-19
CD8	IHC	98941S	Cell Signaling Technology	D4W2Z	6
CD45 FITC	Flow cytometry/WB	103108	BioLegend	30-F11	B388747
CD3 APC-Cy7	Flow cytometry	100222	BioLegend	17A2	B340049
CD4 PerCP-Cy5.5	Flow cytometry	116012	BioLegend	RM4-4	B273144
CD4 BV421	Flow cytometry	100438	BioLegend	GK1.5	B345333
CD8 PerCP	Flow cytometry	100732	BioLegend	53-6.7	B256659
IFN-γ BV510	Flow cytometry	505842	BioLegend	XMG1.2	B294311
TNF-α PE	Flow cytometry	506306	BioLegend	MP6-XT22	B336283
IL4 PE	Flow cytometry	504104	BioLegend	11B11	B320933
IL6 APC	Flow cytometry	504507	BioLegend	MP5-20F3	B320931
IL17A APC	Flow cytometry	506915	BioLegend	TC11-18H10.1	B312893
FOXP3 PE-Cy5	Flow cytometry	15-5773-82	eBioscience	FJK-16s	2275289
Ki67	Flow cytometry	ab15580	abcam	polyclonal	GR3293864-1
PD1 PE	Flow cytometry	109103	BioLegend	RMP1-30	B367713
Tim3 APC	Flow cytometry	119725	BioLegend	RMT3-23	B315334
NK1.1 APC	Flow cytometry	108731	BioLegend	PK136	3295937
Ly6C BV605	Flow cytometry	128036	BioLegend	HK1.4	B335981
CD11b BV650	Flow cytometry	101259	BioLegend	M1/70	B350046
Ly6G APC-Cy7	Flow cytometry	127624	BioLegend	1A8	B348489
F4/80 PerCP-Cy5.5	Flow cytometry	123128	BioLegend	BM8	B256061
CD206 BV421	Flow cytometry	141721	BioLegend	C068C2	B361008
Granzyme B-BV421	Flow cytometry	396414	BioLegend	QA18A28	B369589
Alexa flour 647 anti Rabbit IgG	Flow cytometry	A21244	Invitrogen	Polyclonal	2277746
Goat anti rabbit HRP conjugated	WB	ZB-2301	ZSGB-Bio	Polyclonal	210830707
