# Supplementary material for: B cell lymphoma 6 promotes hepatocellular carcinoma progression by inhibiting tumor infiltrating CD4+T cell cytotoxicity through ESM1
Source: NPJ Precis Oncol. 2024 Jul 1;8:139. doi: 10.1038/s41698-024-00625-7 (PMC11220024; doi:10.1038/s41698-024-00625-7)
Supplement: Supplementary file 2 — Reporting summary [file 41698_2024_625_MOESM2_ESM.pdf]

Corresponding author(s): Cheng Qian

Last updated by author(s): Apr 29, 2024

Reporting Summary

Nature Portfolio wishes to improve the reproducibility of the work that we publish. This form provides structure for consistency and transparency in reporting. For further information on Nature Portfolio policies, see our Editorial Policies and the Editorial Policy Checklist.

Statistics

For all statistical analyses, confirm that the following items are present in the figure legend, table legend, main text, or Methods section.

n/a	Confirmed	
☐	☒	The exact sample size (*n*) for each experimental group/condition, given as a discrete number and unit of measurement
☐	☒	A statement on whether measurements were taken from distinct samples or whether the same sample was measured repeatedly
☐	☒	The statistical test(s) used AND whether they are one- or two-sided<br>*Only common tests should be described solely by name; describe more complex techniques in the Methods section.*
☒	☐	A description of all covariates tested
☒	☐	A description of any assumptions or corrections, such as tests of normality and adjustment for multiple comparisons
☐	☒	A full description of the statistical parameters including central tendency (e.g. means) or other basic estimates (e.g. regression coefficient) AND variation (e.g. standard deviation) or associated estimates of uncertainty (e.g. confidence intervals)
☐	☒	For null hypothesis testing, the test statistic (e.g. *F*, *t*, *r*) with confidence intervals, effect sizes, degrees of freedom and *P* value noted<br>*Give P values as exact values whenever suitable.*
☒	☐	For Bayesian analysis, information on the choice of priors and Markov chain Monte Carlo settings
☒	☐	For hierarchical and complex designs, identification of the appropriate level for tests and full reporting of outcomes
☐	☒	Estimates of effect sizes (e.g. Cohen's *d*, Pearson's *r*), indicating how they were calculated

Our web collection on statistics for biologists contains articles on many of the points above.

Software and code

Policy information about availability of computer code

Data collection	For the analysis of transcriptional factors involved in T cells infiltration from TCGA data, we used TCGA biolinks package version 2.30.0
Data analysis	For the analysis of transcriptional factors involved in T cells infiltration from TCGA data, we used DESeq2 package version 1.42.1, org.Hseg.db version 3.18.0, ClusterProfiler package version 4.8.3, enrichplot package version 1.22.0, ggplot2 package version 3.5.0, R package version 4.3.1, Homer version 4.10. For single cell RNA sequencing data analysis, we used CellRanger version 2.2.0, DoubletFinder version 2.0.3, Harmony package version 0.1.0.

For manuscripts utilizing custom algorithms or software that are central to the research but not yet described in published literature, software must be made available to editors and reviewers. We strongly encourage code deposition in a community repository (e.g. GitHub). See the Nature Portfolio guidelines for submitting code & software for further information.

Data

Policy information about availability of data

All manuscripts must include a data availability statement. This statement should provide the following information, where applicable:

- Accession codes, unique identifiers, or web links for publicly available datasets
- A description of any restrictions on data availability
- For clinical datasets or third party data, please ensure that the statement adheres to our policy

All relevant data are available on request from the corresponding authors (Cheng Qian, email:cqian3184@163.com Juanjuan Shan, email:juanjuansh@gmail.com).

The single cell RNA sequencing raw data for Bcl6 knockout and control H22 derived tumors has been deposited in Sequence Read Archive (SRA) dataset (Accession code: PRJNA1092723). RNA sequencing raw data for H22 wild type cell line and Bcl6 knockout cell line has been deposited in Sequence Read Archive (SRA) data set (Accession code: PRJNA1092336).

Research involving human participants, their data, or biological material

Policy information about studies with human participants or human data. See also policy information about sex, gender (identity/presentation), and sexual orientation and race, ethnicity and racism.

Reporting on sex and gender	The HCC liver samples used in this study contain both male and female samples. The mechanism about ESM1-BCL6 correlation seems to be more significant in male but not in female.
Reporting on race, ethnicity, or other socially relevant groupings	The race, ethnicity, or other socially relevant groupings were not considered in this study.
Population characteristics	This study only involve liver cancer patients tissue sample, the population characteristics is not considered.
Recruitment	Not applicable.
Ethics oversight	the Ethics Committee of Chongqing University Cancer Hospital

Note that full information on the approval of the study protocol must also be provided in the manuscript.

Field-specific reporting

Please select the one below that is the best fit for your research. If you are not sure, read the appropriate sections before making your selection.

☒ Life sciences ☐ Behavioural & social sciences ☐ Ecological, evolutionary & environmental sciences

For a reference copy of the document with all sections, see nature.com/documents/nr-reporting-summary-flat.pdf

Life sciences study design

All studies must disclose on these points even when the disclosure is negative.

Sample size	Sample size were determined according to the animal ethics and published research references. All the data was labled n number for each group.
Data exclusions	No data was excluded in this study.
Replication	All experiments in this study were repeated 3 times at least to confirm the result of each experiment.
Randomization	All allocations in this study is randomized.
Blinding	For the treatment of the mice with anti-PD1, the allocation for the treatment of IgG control and anti-PD1 administration is blinding for this study.

Reporting for specific materials, systems and methods

We require information from authors about some types of materials, experimental systems and methods used in many studies. Here, indicate whether each material, system or method listed is relevant to your study. If you are not sure if a list item applies to your research, read the appropriate section before selecting a response.

Materials & experimental systems

n/a	Involved in the study	
☐	☒	Antibodies
☐	☒	Eukaryotic cell lines
☒	☐	Palaeontology and archaeology
☐	☒	Animals and other organisms
☒	☐	Clinical data
☒	☐	Dual use research of concern
☒	☐	Plants

Methods

n/a	Involved in the study	
☒	☐	ChIP-seq
☐	☒	Flow cytometry
☒	☐	MRI-based neuroimaging

Antibodies

Antibodies used	The antibody information was provided in the supplementary materials.
Validation	The antibody used in this study were validated by the manufacturers.

Eukaryotic cell lines

Policy information about cell lines and Sex and Gender in Research

Cell line source(s)	Hepa1-6, H22, Hep53.4 and Hep3B HCC cell lines were purchased from Procell Life Science & Technology (Wuhan, China).
Authentication	The authentication of the cell lines used in this study was done by Procell Life Science & Technology (Wuhan, China).
Mycoplasma contamination	All the cell lines were routinely tested negative for Mycoplasma contamination.
Commonly misidentified lines (See ICLAC register)	Not applicable for this study.

Animals and other research organisms

Policy information about studies involving animals; ARRIVE guidelines recommended for reporting animal research, and Sex and Gender in Research

Laboratory animals	BALB/c and C57BL/6N mice were used in this study.
Wild animals	This study did not involve wild animals.
Reporting on sex	For animal studies, this study do not containe sex biased study, in vivo experiment were conducted on male or female mice based on the origin of the cell lines.
Field-collected samples	This study did not contain samples collected from the field.
Ethics oversight	The protocol was approved by the the Ethics Committee of Chongqing University Cancer Hospital.

Note that full information on the approval of the study protocol must also be provided in the manuscript.

Plants

Seed stocks	Not applicable for this study.
Novel plant genotypes	Not applicable for this study.
Authentication	Not applicable for this study.

Flow Cytometry

Plots

Confirm that:

- ☒ The axis labels state the marker and fluorochrome used (e.g. CD4-FITC).
- ☒ The axis scales are clearly visible. Include numbers along axes only for bottom left plot of group (a 'group' is an analysis of identical markers).
- ☒ All plots are contour plots with outliers or pseudocolor plots.
- ☒ A numerical value for number of cells or percentage (with statistics) is provided.

Methodology

Sample preparation	All sample preparation protocols were described accordingly in the manuscript.

Instrument	Beckman CytoflexLX
Software	FlowJo software (Tree star)
Cell population abundance	The cell identity was determined by SSC and FSC value. Cell population percentage was calculated for the comparison for each parameter.
Gating strategy	The detailed gating strategies were provided in the supplementary information. The "negative" and "positive" population was determined by the unstained control sample.

☒ Tick this box to confirm that a figure exemplifying the gating strategy is provided in the Supplementary Information.
